# The effect of sodium-glucose cotransporter 2 inhibitors on biomarkers of inflammation: A systematic review and meta-analysis of randomized controlled trials

**DOI:** 10.3389/fphar.2022.1045235

**Published:** 2022-11-11

**Authors:** Dongmei Wang, Jieying Liu, Ling Zhong, Shunhua Li, Liyuan Zhou, Qian Zhang, Ming Li, Xinhua Xiao

**Affiliations:** ^1^ Department of Endocrinology, NHC Key Laboratory of Endocrinology, Peking Union Medical College Hospital, Peking Union Medical College and Chinese Academy of Medical Sciences, Beijing, China; ^2^ Department of Medical Research Center, Peking Union Medical College Hospital, Chinese Academy of Medical Sciences and Peking Union Medical College, Beijing, China

**Keywords:** SGLT2 inhibitor, inflammation, diabetes, meta-analysis, randomized controlled trial

## Abstract

**Aims:** Inflammatory biomarkers may play vital roles in the pathophysiology of diabetes and diabetic cardiorenal complications. Sodium-glucose cotransporter-2 (SGLT2) inhibitors have a potential cardiovascular and renal protective effect in type 2 diabetes. The aim of this meta-analysis was to quantify the effects of SGLT2 inhibitors on biomarkers of inflammation in randomized controlled trials (RCTs).

**Methods:** PubMed, Cochrane Library, EMBASE, and Web of Science were searched for eligible RCTs of adults with type 2 diabetes (T2D) with no time limit (updated to 12 October 2022). The biomarkers selected included C-reactive protein (CRP), interleukin-6, tumor necrosis factor-alpha, leptin, adiponectin, ferritin, plasminogen activator inhibitor (PAI)-1, and vascular cell adhesion molecule-1. Data were analyzed using a random-effect model in Review Manager 5.4.

**Results:** Thirty-four studies with 6,261 patients (68.6% male) were eligible for this meta-analysis. The mean age of the participants was 62.57(±11.13) years old, and the median treatment duration length with follow-up was 24 weeks. Generally, the included trials were of good methodological quality. The meta-analysis revealed that ferritin levels were significantly reduced in SGLT2 inhibitor treatment groups *versus* placebo or standard diabetes therapies (SMD: −1.21; 95% CI: −1.91, −0.52, *p* < 0.001). The effects of CRP (SMD: 0.25; 95% CI: −0.47, −0.03, *p* = 0.02) and leptin (SMD: −0.22; 95% CI: −0.43, −0.01, *p* = 0.04) were reduced, and the effects of adiponectin were improved (SMD: 0.28; 95% CI: 0.15, 0.41, *p* < 0.001) in placebo-controlled studies. PAI-1 levels were significantly reduced in studies controlled for diabetes therapies (SMD: −0.38; 95% CI: −0.61, −0.15, *p* = 0.001).

**Conclusion:** This analysis provides strong evidence supporting anti-inflammatory effects of SGLT2 inhibitors in T2D subjects. The mechanisms and possible targets for the inflammation reducing and cardiorenal protective properties of SGLT2 inhibitors remain to be explored.

## Introduction

There has been a dramatic increase in the prevalence of type 2 diabetes (T2D) in recent years that has reached epidemic proportions (Zheng et al., 2018). Diabetic hyperglycemia often causes both macrovascular and microvascular pathological changes, resulting in increased cardiovascular disease (CVD), diabetic nephropathy risks, and reduced survival rates (Braunwald, 2019). Chronic inflammation in T2D is widely believed to be closely linked to the onset and progression of cardiorenal dysfunction (Luc et al., 2019; Rohm et al., 2022). Hyperglycemia-related diabetic CVD and retinopathy have been reported to be accompanied by the activation of proinflammatory biomarkers, such as C-reactive protein (CRP), interleukin-6 (IL-6), and tumor necrosis factor-alpha (TNF-α) (Qu et al., 2014; Feigerlová and Battaglia-Hsu, 2017; Khaloo et al., 2020). Two adipokines, adiponectin and leptin, are vital biomarkers of metabolic disease and CVD (Everson-Rose et al., 2021; Zhao et al., 2021). A high leptin/adiponectin ratio in patients with gestational diabetes mellitus was related to a higher CVD risk profile during follow-up in a prospective cohort study (Lekva et al., 2017). In addition, plasminogen activator inhibitor (PAI)-1 and vascular cell adhesion molecule (VCAM)-1 have been reported to be significantly increased in diabetic vascular disorders (Altalhi et al., 2021; Bilen et al., 2021). Therefore, hypoglycemic intervention may exert its role in the prevention of diabetic cardiorenal complications by inhibiting inflammation (Luc et al., 2019; Altalhi et al., 2021).

Sodium-glucose cotransporter 2 (SGLT2) inhibitors have been approved for T2D treatment since 2012 (Cahn et al., 2021). Several large clinical trials, such as EMPEROR-Reduced, CANVAS-R, and DAPA-HF, have provided significant clinical evidence of cardiac and renal protection in patients with or without diabetes (Neal et al., 2017; Zannad et al., 2020; Packer et al., 2021). Experimental in vitro and in vivo studies have shown anti-inflammation as a possible beneficial mechanism of SGLT2 inhibitors (Winiarska et al., 2021; Marfella et al., 2022; Scisciola et al., 2022). Bray et al. summarized the evidence of the effects of SGLT2 inhibitors on four inflammatory biomarkers (CRP, IL-6, TNF-α, and adiponectin) in a systematic review including literatures searched up to the end of December 2019. However, the impacts of SGLT2 inhibitors on inflammatory biomarkers were inconsistent in the reviewed literature (Bray et al., 2020). In addition to randomized controlled trials (RCTs), they also included observational studies (Bray et al., 2020). In the past three years, many new RCTs studies have been published on the effects of SGLT-2 inhibitors on biomarkers of inflammation (Docherty et al., 2022; Omar et al., 2022; Takahashi et al., 2022). In addition to the abovementioned biomarkers, ferritin, a specific inflammatory indicator, has been found to be significantly reduced in SGLT2 inhibitor-treated participants (Kinoshita et al., 2020; Alshnbari and Idris, 2022; Takahashi et al., 2022). Therefore, in this meta-analysis we mainly focused on RCTs and included more inflammatory biomarkers to assess the comprehensive effects of SGLT2 inhibitors on biomarkers of inflammation.

## Materials and methods

### Literature search strategy

Four electronic databases (PubMed, Cochrane Library, EMBASE, and Web of Science) were searched for eligible studies without any time limit (updated to 12 October 2022). The keywords used in the search were (sodium-glucose cotransporter 2 inhibitor* OR SGLT-2 inhibitor* OR SGLT 2 inhibitor* OR SGLT2 inhibitor* OR gliflozin* OR canagliflozin OR dapagliflozin OR empagliflozin OR ertugliflozin OR ipragliflozin OR licogliflozin OR remogliflozin OR sergliflozin) AND (inflammation OR C-reactive protein OR interleukin-6 OR tumor necrosis factor-alpha OR leptin OR adiponectin OR plasminogen activator inhibitor-1 and vascular cell adhesion molecule-1 OR ferritin) AND randomized controlled trials. The literature search was conducted in accordance with the preferred reporting items for systematic reviews and meta-analyses (PRISMA) guidelines (Supplementary Table S1). A detailed list of the search terms is given in Supplementary Table S2.

### Inclusion criteria and exclusion criteria

We included all peer-reviewed, published English-language papers of individual RCTs of any SGLT2 inhibitor in adults with T2D. RCTs comparing SGLT2 inhibitors to placebo or other hypoglycemic agents were included without restriction of diabetes duration or length of follow-up. Observational studies, duplicate reports, and articles not reporting outcomes of interest or without original data in human participants were excluded. Studies of prediabetics, pregnant women, type 1 diabetics, or participants receiving SGLT2 inhibitors with fixed-dose combinations or comparisons of different SGLT2 inhibitors were also excluded.

### Outcomes of biomarkers of interest

Our primary outcomes were the changes in the levels of inflammatory biomarkers of interest after treatment with any SGLT2 inhibitor compared to placebo or active control. The biomarkers selected were those that are widely accepted or relevant to clinical therapy (Bray et al., 2020; Bray et al., 2021), including CRP, TNF-α, IL-6, leptin, adiponectin, PAI-1, VCAM-1, and ferritin.

### Data extraction and quality assessment

Two researchers (DMW and JYL) searched the literature independently and extracted data independently based on a standardized data extraction process. The detailed data extraction items included: first author, publication year, population, number of participants, treatment duration, study arms (placebo or diabetes medications), sample characteristics, effect sizes, and 95% CIs. The quality of the identified RCTs was assessed using the Review Manager 5.4 (Cochrane Collaboration, Oxford, United Kingdom) risk of bias tool, which has four sections: recruitment bias, baseline imbalance, loss of clusters, and incorrect analysis. The possible answers were low risk of bias, high or unclear according to the Cochrane Handbook (Higgins et al., 2011). The assessment of publication bias was conducted using a funnel plot. Any disagreements that appeared during the data extraction and quality assessment were resolved by consensus or by a third reviewer as needed (XHX).

### Statistical analysis

In the primary analysis we compared biomarkers of inflammation in patients treated with SGLT2 inhibitors with those receiving placebo or other diabetes medications. The differences in means during the follow-up periods between the treatment and placebo groups was calculated using standardized mean differences (SMDs) with 95% confidence intervals (CIs). Data were analyzed using a random-effect model. If multiple doses or multiple control groups were reported in the same study, a weighted average was taken and combined (Bray et al., 2021). If the mean and SD were not presented, SD was determined from the standard error of the mean, or values were estimated from the sample size, median, range, and/or interquartile range using methodology from the Cochrane Handbook for Systematic Reviews of Interventions (version 5.1.0). Heterogeneity across studies was assessed by the *I* ^*2*^ statistic.

## Results

### Search results and description of the included studies

**FIGURE 1:**
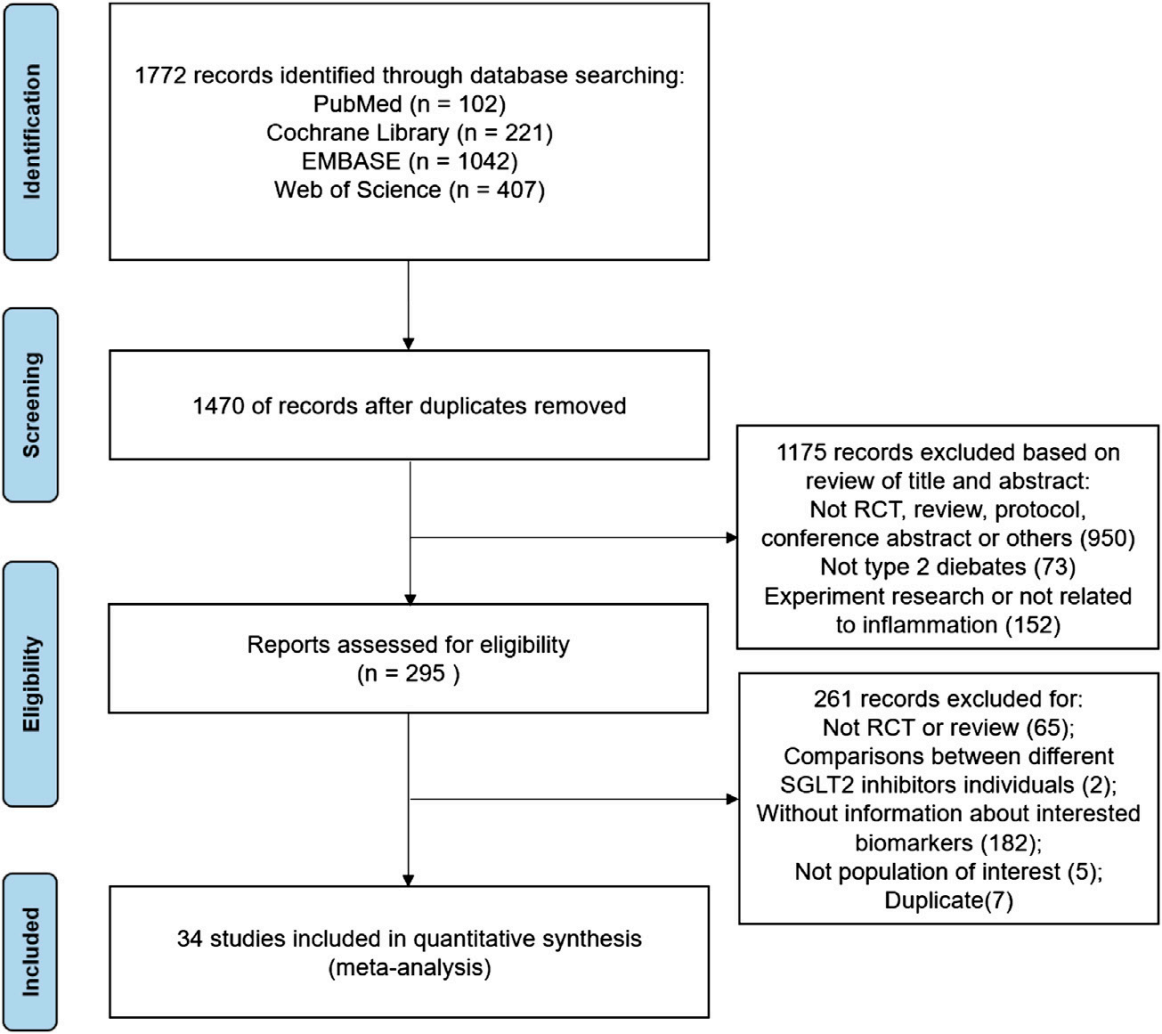
The process of study selection.

The PRISMA flow chart summarizes the search and study selection process (Figure 1). The electronic search yielded 1,772 unique records, of which 295 were reviewed in full text. In total, thirty-four RCTs, comprising 6,261 patients (68.64% male), were included in the final meta-analysis. Of the 34 trials from which data were used for this analysis, most of the studies were of a parallel design, except for two crossover designs. Except for three multinational trials (Garvey et al., 2018; Oldgren et al., 2021; Docherty et al., 2022), most trials were recruited from ten different countries: 17 from **Japan** (Kaku et al., 2014; Kashiwagi et al., 2015a; Kashiwagi et al., 2015b; Kashiwagi et al., 2015c; Ishihara et al., 2016; Ito et al., 2017; Shigiyama et al., 2017; Hattori, 2018; Sato et al., 2018; Seino et al., 2018; Aso et al., 2019; Koshizaka et al., 2019; Katakami et al., 2020; Kinoshita et al., 2020; Sakurai et al., 2020; Ejiri et al., 2022; Takahashi et al., 2022); three from **Germany** (Bosch et al., 2019; Kahl et al., 2020; Thiele et al., 2021); two from **Denmark** (Eickhoff et al., 2020; Omar et al., 2022); two from the **United Kingdom** (Bailey et al., 2012; Brown et al., 2020); two from **Thailand** (Phrommintikul et al., 2019; Phrueksotsai et al., 2021); one from **China** (Hao et al., 2022); one from **Austria** (Antlanger et al., 2022); one from **Brazil** (Sposito et al., 2021); one from **Finland** (Latva-Rasku et al., 2019); and one from the **Netherlands** (Dekkers et al., 2018)). The study characteristics are listed in Table 1. The median length of follow-up was 24 weeks. The mean participant age was 62.57(±11.13) years old, the mean body mass index was 28.23(±5.39) kg/m^2^, the mean HbA1c (%) was 8.02(±4.64), and the mean diabetes duration was 8.27(±7.39) years.

**TABLE 1:** Main characteristics of patients included in the meta-analysis.

Trials	No. of participants	Treatment duration	Population	Study arms	Age (year)	No. (%) of male	BMI (kg/m^2^)	HbA1c (%)	Diabetes duration (year)
Docherty et al., 2022	2423	12 months	T2D, multiple countries	Dapagliflozin 10 mg/day; placebo	67.2 ± 10.4	- (77.9)	28.5 ± 5.9	-	-
Ejiri et al., 2022	157	12 weeks	T2D with heart failure, Japan	Luseogliflozin 2.5 mg/day; voglibose	71.8 ± 7.8; 74.9 ± 7.6	54 (68.4); 45 (57.7)	25.4 ± 4.4; 25.1 ± 4.2	7.0 ± 0.7; 6.9 ± 0.8	72 (24–130)^#^; 72 (36–138)^#^
Takahashi et al., 2022	50	72 weeks	T2D with NAFLD, Japan	Ipragliflozin 50 mg/day; antidiabetic drugs	59.0 (46.8–64.3); 50.0 (48.0–68.8)	15 (62.5); 14 (53.8)	29.9 (27.2–32.3); 28.8 (25.7–32.9)	6.5 (6.1–7.1); 6.8 (6.3–7.0)	-
Omar et al., 2022	187	12 weeks	T2D with stable HFrEF, Denmark	Empagliflozin, 10 mg/day; placebo	65 ± 10; 63 ± 12	78 (83); 81 (87)	29 (26–32); 28 (26–33)	40 (36–43)^a^; 39 (36–42)^a^	-
Hao et al., 2022	142	12 weeks	Newly-diagnosed T2D, China	Canagliflozin 100 mg/day; metformin	57.3 ± 9.8; 56.0 ± 8.5	36 (52.2); 39 (53.4)	26.4 ± 5.0; 26.3 ± 4.8	7.9 ± 0.4; 8.0 ± 0.4	less than 6 months
Antlanger et al., 2022	23	12 weeks	T2D with CKD stages 3–4, Austria	Empagliflozin 10 mg/day; placebo	71 ± 6; 69 ± 12	10 (91.6); 7 (58.3)	31 ± 3.9; 28 ± 5.5	6.9 ± 1.3; 7 ± 1.1	14.3 ± 9.5
Sposito et al., 2021	97	12 weeks	T2D with high CVD risk, Brazil	Dapagliflozin 10 mg/day; glibenclamide	57 ± 7; 58 ± 7	29 (60); 30 (61)	31 ± 4; 30 ± 5	7.9 ± 0.9; 7.9 ± 0.9	9 ± 7; 10 ± 7
Phrueksotsai et al., 2021	38	12 weeks	T2D with NAFLD, Thailand	Dapagliflozin 10 mg/day; placebo	57.0 ± 6.9; 61.2 ± 7.2	5 (27.8); 7 (35)	29.6 ± 4.0; 28.8 ± 4.1	8.2 ± 0.8; 7.8 ± 0.6	4.5 (2–10); 5.5(2.5–10)
Oldgren et al., 2021	49	6 weeks	T2D, Sweden and Finland	Dapagliflozin 10 mg/day; placebo	63.5 ± 7.9; 65.4 ± 6.5	9 (37.5); 17 (68)	30.2 ± 3.6; 30.1 ± 3.8	6.74 ± 0.58; 6.67 ± 0.65	5.3 (0.6–16.3);
									3.8 (0.7–27.8)
Thiele et al., 2021	42	3 months	T2D, Germany	Empagliflozin 10 mg; placebo	62.8 ± 5.4; 61.2 ± 7.9	16 (80); 18 (81.8)	31.4 ± 5.3; 31.2 ± 4.0	7.8 ± 1.5; 7.9 ± 1.3	10 (4–14) 9(6–16)
Kaku et al., 2014	235	24 weeks	T2D, Japan	Tofogliflozin 10 mg/day; tofogliflozin 20 mg/day; tofogliflozin 40 mg/day; placebo	58.6 ± 9.8; 56.6 ± 10.2; 57.0 ± 9.1; 56.8 ± 9.9	38 (66.7); 39 (67.2); 39 (67.2); 37 (66.1)	25.07 ± 3.53; 24.99 ± 4.55; 25.78 ± 4.10; 26.00 ± 4.11	8.45 ± 0.75; 8.34 ± 0.81; 8.37 ± 0.77; 8.41 ± 0.78	6.3 ± 7.1; 6.4 ± 5.1; 6.7 ± 5.5; 6.0 ± 6.1
Sakurai et al., 2020	49	12 weeks	T2D, Japan	Empagliflozin 10 mg/day; standard therapy	58.6 ± 12.9;	16 (51.6);	27.2 ± 5.7;	8.00 ± 0.86;	-
					58.6 ± 12.2	12 (66.7)	28.4 ± 6.6	7.63 ± 0.55	
Kinoshita et al., 2020	98	28 weeks	T2D with NAFLD, Japan	Dapagliflozin 5 mg/day; pioglitazone; glimepiride	58.7 ± 1.6;	15 (46.9);	29.5 ± 0.8;	7.4 ± 0.2;	6.6 ± 0.9;
					59.0 ± 1.9	15 (45.5);	28.7 ± 0.9;	7.4 ± 0.2;	7.9 ± 0.8;
					58.0 ± 2.3;	15 (45.5)	28.4 ± 0.7	7.6 ± 0.2	7.2 ± 1.0
Katakami et al., 2020	339	104 weeks	T2D, Japan	Tofogliflozin 20 mg/day;	61.3 ± 9.3;	99 (58.6);	27.0 ± 5.8;	7.4 ± 0.7;	12.1 ± 8.4;
				Conventional treatment	60.9 ± 9.7	99 (58.2)	27.0 ± 4.6	7.3 ± 0.7	12.5 ± 8.3
Kahl et al., 2020	84	24 weeks	T2D, Germany	Empagliflozin 25 mg/day; placebo	62.7 ± 7.0;	29 (69);	32.1 ± 4.6;	6.8 ± 0.5;	36 ± 27^#^;
					61.5 ± 10.0	29 (69)	32.4 ± 4.2	6.7 ± 0.7	40 ± 27^#^
Eickhoff et al., 2020	36	12 weeks	T2D with albuminuria, Denmark	Dapagliflozin 10 mg; placebo	64 ± 8	32 (89)	32.8 ± 5.7	8.9 ± 1.4	16.4 ± 4.7
Brown et al., 2020	66	12 months	T2D with LVH, UK	Dapagliflozin 10 mg/day;	64.25 ± 7.01;	20 (62.5);	32.30 ± 4.66;	61.75 ± 11.19^a^;	8.5 (5.25, 14.5);
				Placebo	66.74 ± 6.62	18 (52.9)	32.59 ± 4.22	60.18 ± 10.15^a^	10.0 (7.5, 15.0)
Phrommintikul et al., 2019	49	24 weeks	T2D, Thailand	Dapagliflozin 10 mg/day; vildagliptin	62.60 ± 8.27;	14 (56);	25.63 ± 3.00;	8.17 ± 1.41;	-
					63.88 ± 7.65	12 (50)	24.90 ± 3.16	8.25 ± 1.13	
Latva-Rasku et al., 2019	31	8 weeks	T2D, Finland	Dapagliflozin 10 mg/day; placebo	62 ± 8.4;	13 (86.7);	32.1 ± 3.9;	7.0 ± 0.6;	7.8 ± 3.8;
					60 ± 7.4	12 (75)	31.7 ± 5.0	6.8 ± 0.5	7.3 ± 3.7
Koshizaka et al., 2019	98	24 weeks	T2D, Japan	Ipragliflozin 50 mg/day; metformin	56.6 ± 11.9;	31 (64.6);	27.55 ± 4.24;	7.95 ± 0.73;	5.4 ± 4.6;
					55.7 ± 12.2	28 (56.0)	28.83 ± 5.32	8.12 ± 0.90	5.3 ± 4.8
Bosch et al., 2019	58	6 weeks	T2D, Germany	Empagliflozin 25 mg/day; placebo	62 ± 7	34 (59)	29.5 ± 3.9	6.69 ± 0.82	-
Aso et al., 2019	57	24 weeks	T2D with NAFLD, Japan	Dapagliflozin 5 mg/day; standard treatment	56.2 ± 11.5;	19 (57.6);	27.6 ± 4.7;	8.37 ± 1.48;	-
					57.1 ± 13.8	15 (62.5)	28.3 ± 3.5	7.70 ± 1.24	
Seino et al., 2018	233	52 weeks	T2D, Japan	Luseogliflozin 2.5 mg/day; placebo	57.4 ± 10.3;	112 (70.4);	25.42 ± 3.53;	8.70 ± 0.83;	11.7 ± 7.6;
					57.1 ± 10.9	51 (68.9)	25.15 ± 3.44	8.84 ± 0.83	12.1 ± 6.8
Hattori, 2018	102	1 year	T2D, Japan	Empagliflozin 10 mg/day; placebo	57.4 ± 12.3;	38 (74.5);	31.0 ± 4.8;	7.01 ± 1.1;	-
					58.1 ± 9.71	41 (80.4)	30.0 ± 4.4	6.84 ± 0.85	
Sato et al., 2018	40	6 months	T2D with CAD, Japan	Dapagliflozin Conventional therapy	68 ± 4;	16 (80);	26.6 ± 4.6;	7.2 ± 0.6;	-
					66 ± 6	14 (70)	25.0 ± 3.1	7.4 ± 1.1	
Garvey et al., 2018	200	52 weeks	T2D, multiple countries	Canagliflozin 300 mg/day glimepiride	58.5 ± 9.0;	48 (48);	32.5 ± 4.7;	7.8 ± 0.9;	7.5 ± 6.0;
					57.5 ± 8.6	55 (55)	31.7 ± 5.0	7.7 ± 0.8	6.8 ± 4.9
Dekkers et al., 2018	31	6 weeks	T2D with albuminuria, the Netherlands	Dapagliflozin 10 mg/day; placebo	62 ± 8.1	24 (77.4)	31 ± 5.4	56 ± 8.5^a^	-
Shigiyama et al., 2017	74	16 weeks	T2D, Japan	Dapagliflozin 5 mg/day; metformin	57.9 ± 8.3;	25 (67.6);	26.8 ± 4.6;	6.8 ± 0.5;	5.4 ± 4.4;
					59.4 ± 10.1	22 (59.5)	26.3 ± 3.5	6.9 ± 0.5	6.3 ± 4.2
Ito et al., 2017	66	24 weeks	T2D with NAFLD, Japan	Lpragliflozin 50 mg/day; pioglitazone	57.3 ± 12.1;	14 (44);	30.7 ± 5.0;	8.5 ± 1.5;	8.7 ± 5.8;
					59.1 ± 9.8	18 (53)	29.9 ± 6.2	8.3 ± 1.4	9.5 ± 5.8
Ishihara et al., 2016	255	16 weeks	T2D, Japan	Ipragliflozin 50 mg/day; placebo	58.7 ± 11.1;	105 (62.5);	25.61 ± 3.53;	8.67 ± 0.77;	151.1 ± 93.5^#^;
					59.2 ± 9.3	51 (58.6)	26.42 ± 3.81	8.62 ± 0.86	171.4 ± 102.5^#^
Kashiwagi et al., 2015a	151	24 weeks	T2D, Japan	Ipragliflozin 50 mg/day; placebo	56.2 ± 10.22;	75 (77.3);	27.11 ± 3.85;	8.24 ± 0.67;	76.0 ± 56.57^#^;
					56.1 ± 11.91	37 (68.5)	27.13 ± 4.31	8.39 ± 0.64	92.5 ± 63.87^#^
Kashiwagi et al., 2015b	129	16 weeks	T2D, Japan	Ipragliflozin 50 mg/day	60.6 ± 9.4;	42 (67.7);	25.3 ± 3.1;	8.40 ± 0.86;	90.4 ± 82.6^#^;
				Placebo	58.3 ± 10.5	48 (71.6)	25.6 ± 3.9	8.25 ± 0.68	70.8 ± 61.1^#^
Kashiwagi et al., 2015c	240	24 weeks	T2D, Japan	Ipragliflozin 50 mg/day; placebo	59.6 ± 10.02;	111 (67.3);	25.81 ± 3.60;	8.38 ± 0.64;	123.8 ± 84.99^#^;
					59.8 ± 8.58	47 (62.7)	24.18 ± 2.97	8.34 ± 0.73	129.0 ± 74.92^#^
Bailey et al., 2012	282	24 weeks	T2D, UK	Dapagliflozin 1 mg/day; dapagliflozin 2.5 mg/day; dapagliflozin 5 mg/day; placebo	53.7 ± 9.04;	38 (52.8);	32.53 ± 5.68;	7.8 ± 0.98;	1.6 ± 2.55;
					53.5 ± 10.61;	34 (45.9);	31.13 ± 5.47;	8.1 ± 1.07;	1.5 ± 2.19;
					51.3 ± 11.51;	32 (47.1);	30.97 ± 5.68;	7.9 ± 1.03;	1.4 ± 3.24;
					53.5 ± 11.08	37 (54.4)	32.47 ± 4.91	7.8 ± 1.12	1.1 ± 1.95

T2D, type 2 diabetes; NAFLD, non-alcoholic fatty liver disease; HFrEF, heart failure with reduced ejection fraction; CKD, chronic kidney disease; CVD, cardiovascular disease; LVH, left ventricular hypertrophy; BMI, body mass index.

^a^ HbA1c (mmol/mol); #diabetes duration (months).

### Risk of bias and quality assessment

**FIGURE 2:**
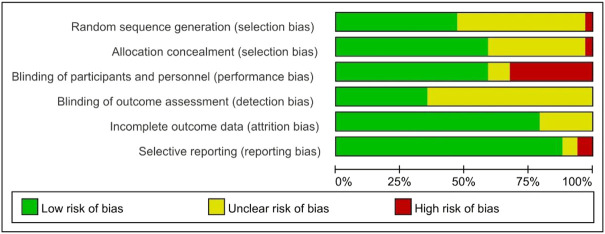
Summary of risk of bias using the Cochrane risk of bias tool.

The methods for risk-of-bias and quality assessment are shown in Figure 2 and Supplementary Figure S1. Due to a lack of detailed methods, 31.4% of assessments across all domains were “unclear risk of bias”. Ten of the 34 studies were judged to have a “high risk of bias” for one domain. Two studies were assessed as having a “high risk of bias” for selection bias and performance bias. With the exception of ten open-label studies, 68.8% of studies reported double-blinding, but most did not describe the steps taken to ensure double-blinding. The majority of included studies were assessed as having a “low risk of bias” for at least four domains.

### Meta-analysis results

#### CRP

**FIGURE 3:**
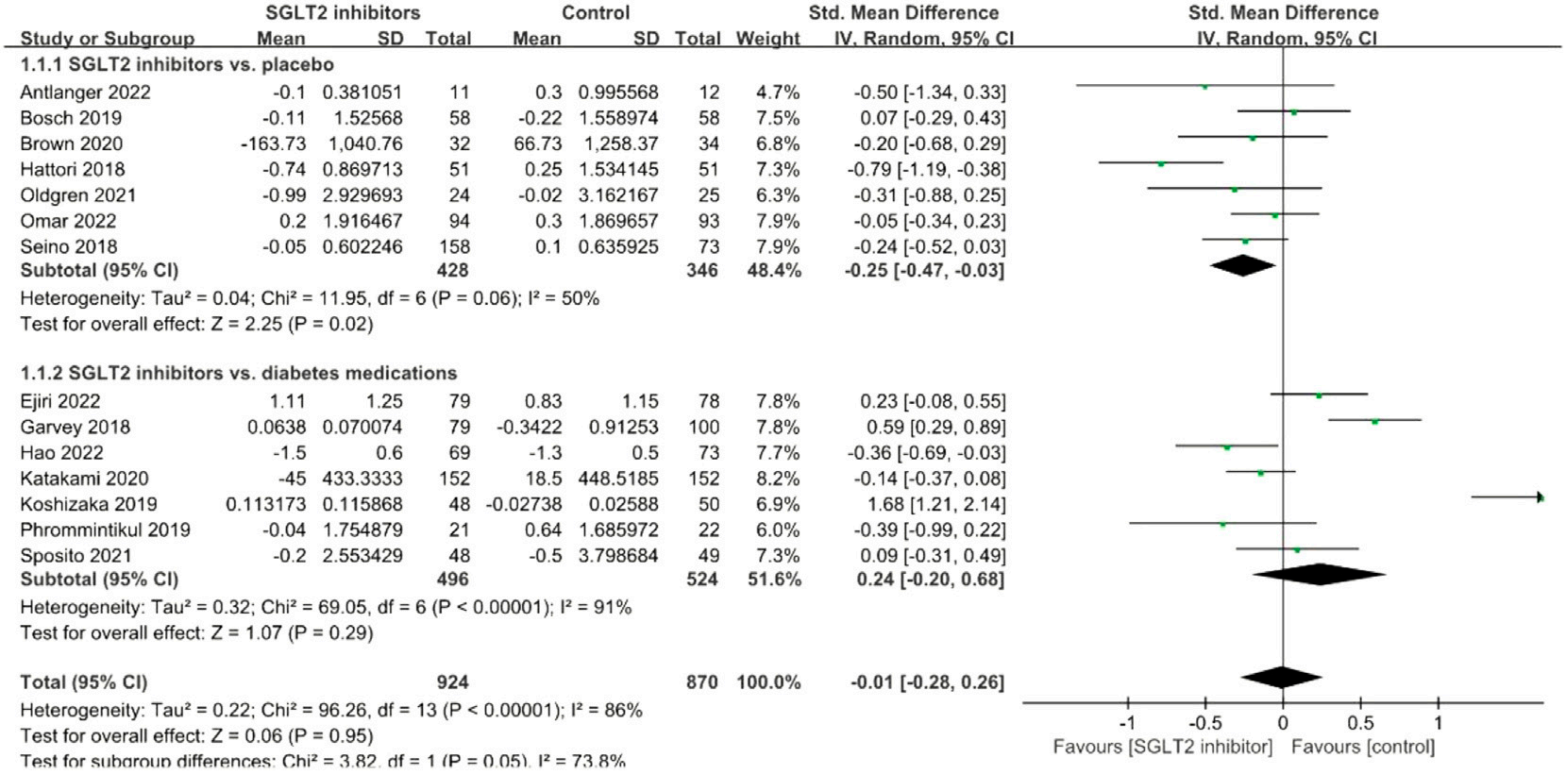
Forest plot of the effects of SGLT2 inhibitors on C-reactive protein.

The pooled effect size of 14 eligible trials (1794 subjects) demonstrated that there was no significant reduction in CRP concentrations (SMD: −0.01; 95% CI: −0.28, 0.26, *p* = 0.95), with considerable heterogeneity between studies (*I* ^*2*^ = 86%, *p* < 0.001; Figure 3). Subgroup analyses were performed based on study arms and showed a significant CRP-lowering effect in placebo-controlled studies (SMD: −0.25; 95% CI: −0.47, −0.03, *p* = 0.02) but not in diabetes medication-controlled studies (SMD: 0.24; 95% CI: −0.20, 0.68, *p* = 0.29). In addition, the source of heterogeneity seemed to be mainly from the diabetes medications-controlled subgroup.

#### TNF-α

**FIGURE 4:**
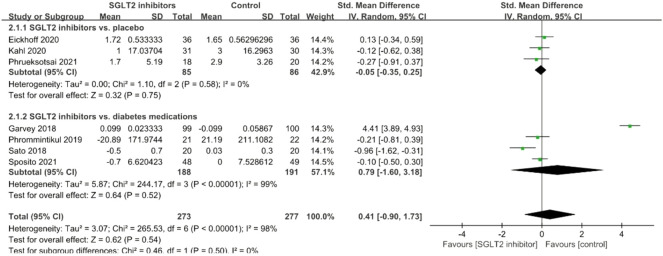
Forest plot of the effects of SGLT2 inhibitors on tumor necrosis factor-alpha.

A total of seven studies explored the effects of SGLT2 inhibitors on serum TNF-α levels, and no significant effect was observed (SMD = 0.41; 95% CI: −0.90, 1.73, *p* = 0.54); but there was significant between-study heterogeneity (*I* ^*2*^ = 98%, *p* < 0.001; Figure 4). Subgroup analyses did not significantly improve the results. After removal of the study conducted by Garvey et al., the *I* ^*2*^-value was reduced from 98% to 33%.

#### IL-6

**FIGURE 5:**
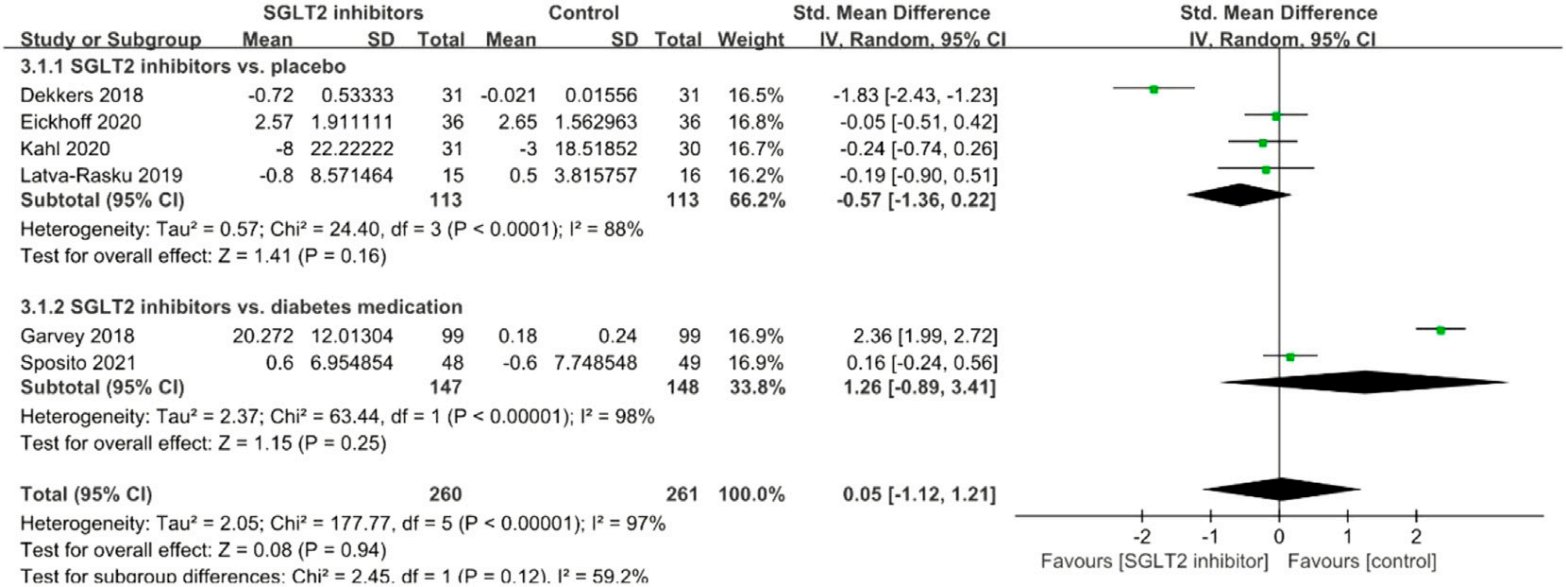
Forest plot of the effects of SGLT2 inhibitors on interleukin-6.

A total of six studies investigated the effects of SGLT2 inhibitors on serum IL-6, and meta-analysis results showed no significant effect (SMD = 0.05; 95% CI: −1.12, 1.21, *p* = 0.94); but there was significant between-study heterogeneity (*I* ^*2*^ = 97%, *p* < 0.001; Figure 5). Subgroup analyses did not significantly improve the results, and removal of any study did not significantly improve heterogeneity.

#### Adiponectin

**FIGURE 6:**
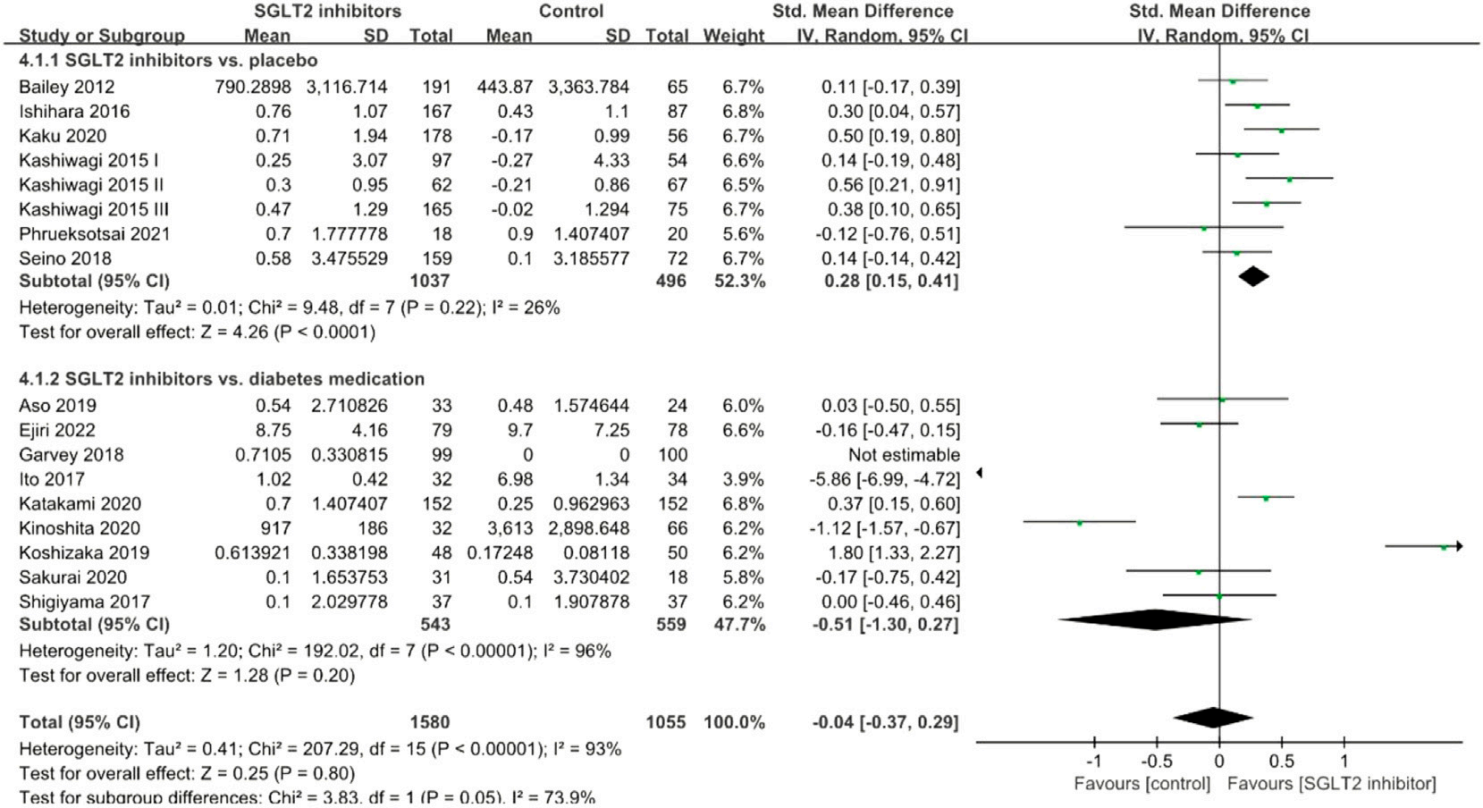
Forest plot of the effects of SGLT2 inhibitors on adiponectin.

Seventeen studies investigated the levels of adiponectin following SGLT2 inhibitor intervention. Figure 6 shows the effects of SGLT2 inhibitors on serum adiponectin levels and our analysis demonstrated no significant effect (SMD = −0.04; 95% CI: −0.37, 0.29, *p* = 0.80; Figure 6). Subgroup analyses revealed a significant adiponectin-improving effect in placebo-controlled studies (SMD: 0.28; 95% CI: 0.15, 0.41, *p* < 0.001) with no heterogeneity, but it was not significantly reduced in diabetes medications-controlled studies (SMD: −0.51; 95% CI: −1.30, 0.27, *p* = 0.20). The source of heterogeneity seemed to be from the diabetes medication-controlled subgroup.

#### Leptin

**FIGURE 7:**
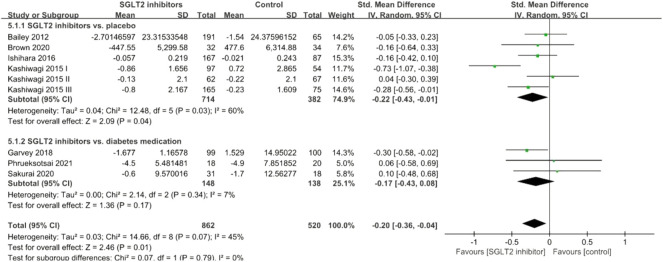
Forest plot of the effects of SGLT2 inhibitors on leptin.

A total of nine studies investigated the effects of SGLT2 inhibitors on serum leptin levels, and the pooled effect size demonstrated that SGLT2 inhibitors had a significant reduction effect (SMD: −0.20; 95% CI: −0.36, −0.04, *p* = 0.01) with slight heterogeneity between studies (*I* ^*2*^ = 45%, *p* = 0.07; Figure 7). Subgroup analyses revealed a significant leptin-lowering effect in placebo-controlled studies (SMD: −0.22; 95% CI: −0.43, −0.01, *p* = 0.04), but not in diabetes medications-controlled studies (SMD: −0.17; 95% CI: −0.43, 0.08, *p* = 0.17).

#### Ferritin

**FIGURE 8:**
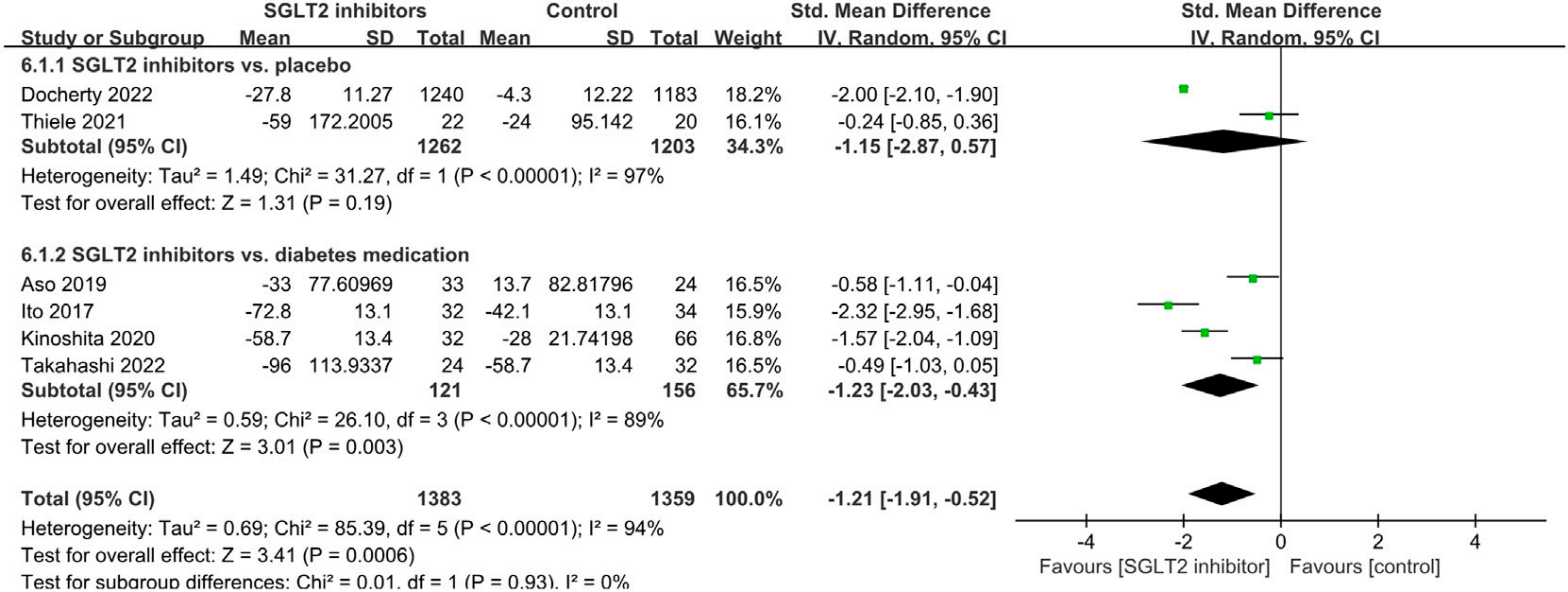
Forest plot of the effects of SGLT2 inhibitors on ferritin.

A total of six studies investigated the effects of SGLT2 inhibitors on serum ferritin levels, and the pooled effect size demonstrated that SGLT2 inhibitors had a significant reduction effect (SMD: −1.21; 95% CI: −1.91, −0.52, *p* < 0.001) with *I* ^*2*^ = 94%, suggesting a high level of heterogeneity among the studies (Figure 8). Subgroup analyses revealed a significant ferritin-lowering effect in diabetes medication-controlled studies (SMD: −1.23; 95% CI: −2.03, −0.43, *p* = 0.003), while only one placebo-controlled study showed no significant reduction (SMD: −1.15; 95% CI: −2.87, 0.57, *p* = 0.19).

#### PAI-1 and VCAM-1

**FIGURE 9:**
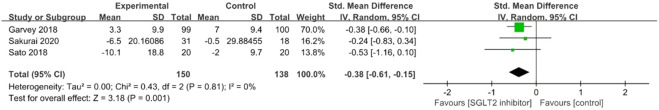
Forest plot of the effects of SGLT2 inhibitors on plasminogen activator inhibitor-1.

A total of three studies investigated the effects of SGLT2 inhibitors on serum PAI-1 levels, and the pooled effect size demonstrated that SGLT2 inhibitors had a significant reduction effect (SMD: −0.38; 95% CI: −0.61, −0.15, *p* = 0.001) with no heterogeneity between studies (*I* ^*2*^ = 0%, *p* = 0.81; Figure 9). However, these three RCTs were all diabetes medication-controlled studies. In addition, VCAM-1 was not assessed due to inadequate data availability, as only one study was available (Garvey et al., 2018).

#### Subgroup analysis depending on treatment duration

The median length of follow-up was 24 weeks, but there was a large variation (6 weeks–104 weeks). Previous studies have found that many metabolic parameter improvements peaked at about week 24 (Yoshida et al., 2019). Thus, we separated the two subgroups into “0–24 weeks” and “longer than 24 weeks”. With the exception of three biomarkers (IL-6, ferritin, and PAI-1) with too few RCTs, the subgroup analysis results of CRP, TNF-α, adiponectin, and leptin were dependent on treatment duration (Supplementary Figure S2). As shown in Supplementary Figures S2A,B, the significant CRP-lowering effect in placebo-controlled studies seems to be mainly in the “longer than 24 weeks” group, and the timeframe seems to have significant effects on adiponectin and leptin levels (Supplementary Figures S2D,F,G); however, only one RCT was included in the “longer than 24 weeks” group.

#### Sensitivity analysis and publication bias

In our meta-analysis of the effects of SGLT2 inhibitor therapy on the inflammatory biomarkers, CRP, TNF-a, IL-6, leptin, adiponectin, and ferritin, the results did not change significantly after removal of any study from the pooled research, which indicated that our analysis model was stable. No evidence of publication bias was detected in this meta-analysis, as the funnel plot of standard error by effect size was symmetrical (Supplementary Figure S3).

## Discussion

T2D is closely linked to inflammation, primarily as low-grade chronic inflammation (Luc et al., 2019; Rohm et al., 2022). The high risk of CVD in T2D is inextricably associated with the activation of inflammatory markers (Luc et al., 2019; Rohm et al., 2022). For significant cardiovascular and renal benefits, SGLT2 inhibitors are regarded as a promising treatment for type 2 diabetes (Neal et al., 2017; Zannad et al., 2020; Packer et al., 2021; Paolisso et al., 2022). In this systematic review and meta-analysis, we investigated whether the improvement of CVD and renal outcomes of SGLT2 inhibitor treatment was due to inflammation regulation by analyzing 34 RCTs to assess the effect of SGLT2 inhibitors on biomarkers of inflammation. The results indicated that, compared to placebo or active diabetes medications, treatment with SGLT2 inhibitors significantly reduced serum ferritin levels. A reduction in CRP and leptin, and an increase in adiponectin were demonstrated in placebo-controlled studies. PAI-1 levels were significantly reduced in diabetes therapy-controlled studies. Given the lack of inflammatory biomarkers from chief cardiorenal outcome RCTs, these results indicate that anti-inflammatory activities may partially mediate the pleiotropic benefits of SGLT2 inhibitors.

Circulating CRP, IL-6, and TNF-α are classic proinflammatory cytokines. We identified 14 eligible studies reporting CRP levels as an outcome measure, seven reporting IL-6 levels, and six reporting TNF-α levels. It was previously shown that SGLT2 inhibitors were associated with reduction of CRP, IL-6, and TNF-α, and reduction of serum CRP appeared to be independent of improvement in HbA1c (Bray et al., 2020). However, except for one subgroup analysis that reported reduced CRP compared to placebo, SGLT2 inhibitors failed to lower the levels of these inflammatory cytokines in our analysis. Among the 13 studies, the largest study was well-designed, and serum CRP did not significantly vary between the tofogliflozin group and the conventional group (Katakami et al., 2020), while only two studies demonstrated a marked decrease in CRP. Hao et al. (2022) and Hattori (2018) were all open-label studies with a “high risk of bias” for performance bias. Notably, unlike GLP-1RAs, SGLT2 inhibitors increase the availability of glucose in the urinary tract, increasing the risk of urinary tract infections (Li et al., 2017). Previous studies showed that SGLT2 inhibitors were linked to infections of the genitourinary tract in T2D (Li et al., 2017; Dave et al., 2019). However, because of conflicting findings with prior meta-analyses reports, the association with UTIs is still unclear (Wu et al., 2016; Puckrin et al., 2018). From the classic chronic proinflammatory markers in our results, we caution against attaching too much importance to the risk of infections of the genitourinary tract from SGLT2 inhibitors, compared with their pleiotropic beneficial impacts on favorable cardiorenal metabolic profiles (Dave et al., 2019).

SGLT-2 inhibitors have been widely reported to promote body weight loss and decline of adipose insulin resistance (Yoshida et al., 2019). Experimental studies have shown the beneficial effects of SGLT-2 inhibitors on adipose tissue metabolism and inflammation (Xu et al., 2017; Xu et al., 2019). In addition to shifting energy metabolism towards fat utilization, SGLT-2 inhibitors were demonstrated to attenuate obesity-related chronic inflammation by reducing M1-polarized macrophage accumulation and inducing the anti-inflammatory M2 macrophage phenotype within adipose tissue and liver in diet-induced obese mice (Xu et al., 2017; Xu et al., 2019). Adiponectin and leptin were identified as unique ‘secretomes’ mediating inter-organ communication between adipose tissue and the cardiovascular system (Everson-Rose et al., 2021; Zhao et al., 2021). Their associations with cardiovascular disorders are paradoxical, however (Zhao et al., 2021). Upregulation of adiponectin has been linked to suppression of chronic inflammation (Hoong and Chua, 2021). In our results, adiponectin levels were significantly increased in placebo-controlled studies, which are in line with the findings of a previous meta-analysis (Wu et al., 2019). Leptin levels were significantly reduced in placebo-controlled studies, and the underlying mechanism may be due to the alleviation of leptin tolerance reported in findings from obese mice and humans (Perakakis et al., 2021). However, an accurate definition of leptin tolerance and the underlying mechanism awaits further investigation. Evidence has also been found that leptin induces the activation of proinflammatory signaling pathways and the increased synthesis of proinflammatory mediators, thereby resulting in vascular inflammation, endothelial dysfunction and atherosclerosis (La Cava, 2017). Taking these considerations into account, our results also suggest that the protective effects of SGLT2 inhibitors may partly be associated with a reduction in leptin levels (Wu et al., 2019).

Intriguingly, we also found that SGLT2 inhibitors significantly reduced serum ferritin and PAI-1 levels. Serum ferritin has been identified as a biomarker of insulin resistance, liver fat accumulation, and greater CVD risk (Ferrannini et al., 2020; Zhou et al., 2021). However, SGLT2 inhibitor-induced decreases in ferritin levels were also linked to increased erythropoietin levels, and erythropoiesis might be related to the inhibition of inflammatory functions (Thiele et al., 2021), as plasma and tissue PAI-1 levels were increased under pathological conditions (Baumeier et al., 2021). The development of PAI-1 inhibitors may provide a potential treatment for renal and cardiovascular disease (Ha et al., 2009). The underlying mechanism of ferritin and PAI-1 reduction by SGLT2 inhibitors certainly deserves further research.

In this meta-analysis, we report a systematic assessment of the effects of SGLT2 inhibitors on a set of inflammatory biomarkers using a robust, systematic methodology. However, we acknowledge some inherent limitations. First, while most major RCTs, such as EMPEROR-Reduced and CANVAS-R, confirmed the cardiorenal protective effects of SGLT2 inhibitors, they did not follow-up on inflammatory biomarkers as outcomes (Packer et al., 2021). Although we conducted a wide-ranging search using four eligible databases, much of the evidence was from smaller trials, of which one-third of RCTs were assessed to have a “high risk of bias”, especially performance bias. Second, a limiting factor of the present review is a high degree of heterogeneity in the majority of biomarkers, which may be explained by different dosages, various SGLT2 inhibitors or active diabetes medication controls, as well as methodology of the assays and units of measure. It is also important to note that the majority of studies included in this meta-analysis were not matched for inflammation biomarkers at baseline. Third, the median length of follow-up was 24 weeks, but with wide variation (6 weeks–104 weeks). From the subgroup analysis of CRP, adiponectin, and leptin, it seemed that the impact of SGLT2 inhibitors was related to timeframe. However, the number of RCTs for conducting subgroup analysis was limited. Longer intervention RCTs are needed to assess the impact of timeframe on these inflammatory biomarkers. Lastly, the data from clinical studies showed that biomarkers of oxidative stress, closely associated with inflammation, were also higher in individuals with diabetes or diabetic complications (Khaloo et al., 2020; Bokhary et al., 2021). However, RCTs on the effect of SGLT2 inhibitors on oxidative stress are still rare, and this is an important area for future research.

## Conclusion

In conclusion, this systematic review demonstrated that some circulating inflammatory related biomarkers were significantly altered by SGLT2 inhibitors, a finding that is important in understanding the cardiovascular and renal protective properties of SGLT2 inhibitors. Despite the lack of direct evidence from larger cardiorenal outcome trials on inflammatory biomarkers, there are reasons to believe that this meta-analysis offers different perspectives that will be of use in the clinical application of SGLT2 inhibitors. This meta-analysis also provides a promising future for the ongoing development of similar drugs and related anti-inflammatory agents in metabolic disorders.
