# Supplementary material for: The effect of sodium-glucose cotransporter 2 inhibitors on biomarkers of inflammation: A systematic review and meta-analysis of randomized controlled trials
Source: Front Pharmacol. 2022 Nov 11;13:1045235. doi: 10.3389/fphar.2022.1045235 (PMC9717685; doi:10.3389/fphar.2022.1045235)
Supplement: Supplementary file 1 [file DataSheet1.docx]

Supplementary Materials

# Supplementary Figures and Tables

## Supplementary Figures

**Figure S1. Risk of bias summary.**


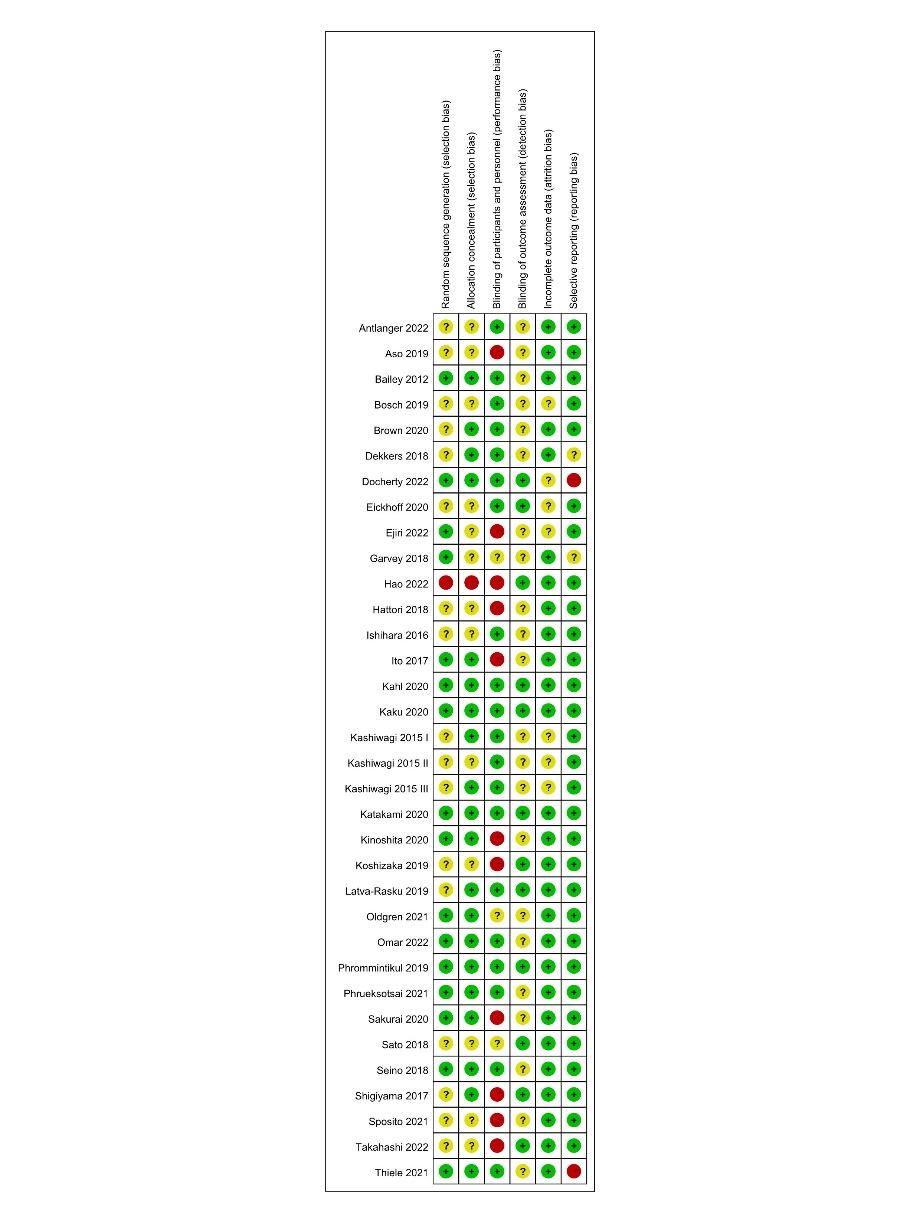


**Figure S2. Forest plot of subgroup analysis depending on the treatment durations.**

1. **C-reactive protein: SGLT2 inhibitors vs. placebo**


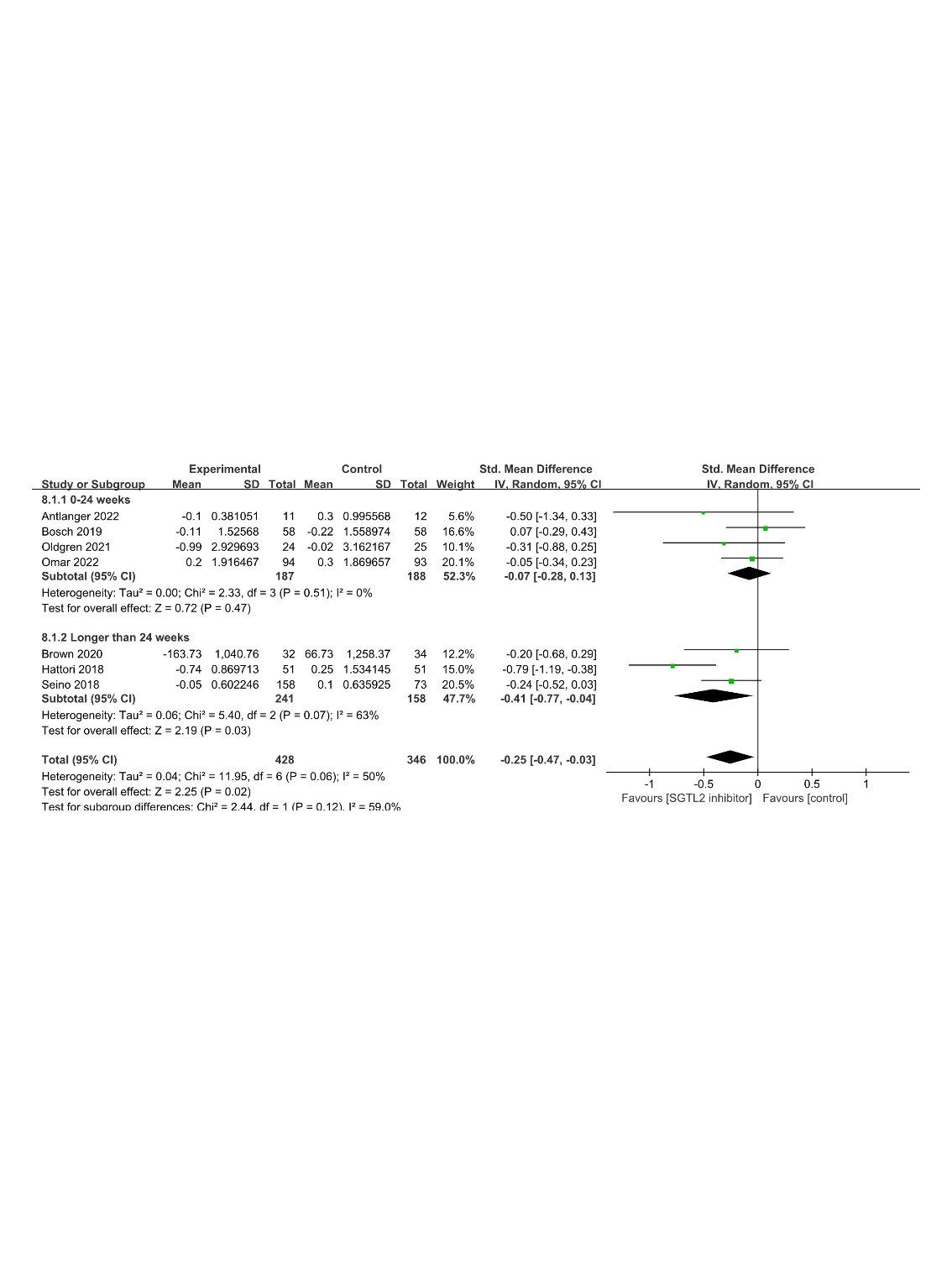


1. **C-reactive protein: SGLT2 inhibitors vs. diabetes medications**


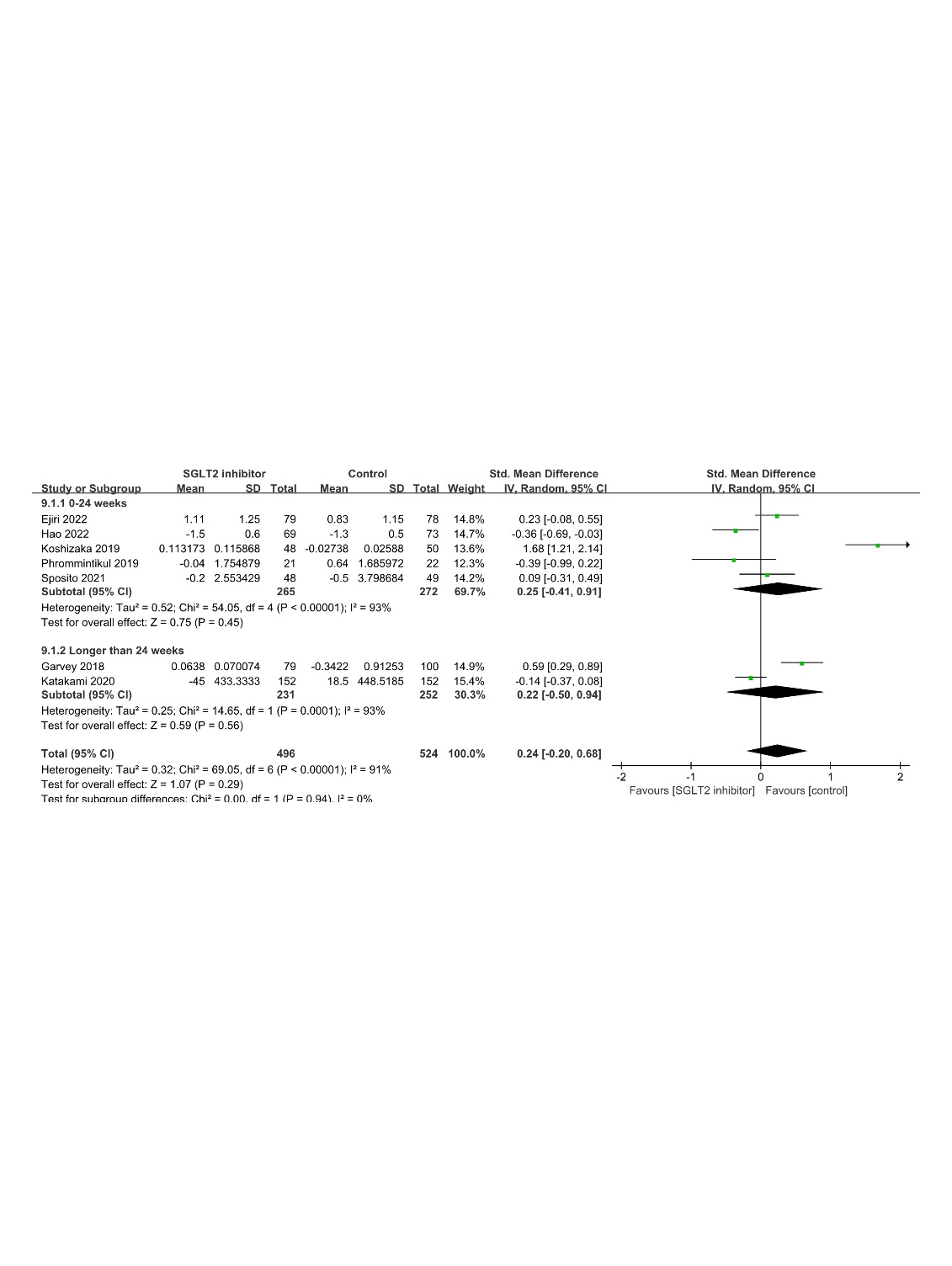


**(C) Tumor necrosis factor-alpha: SGLT2 inhibitors vs. diabetes medications**


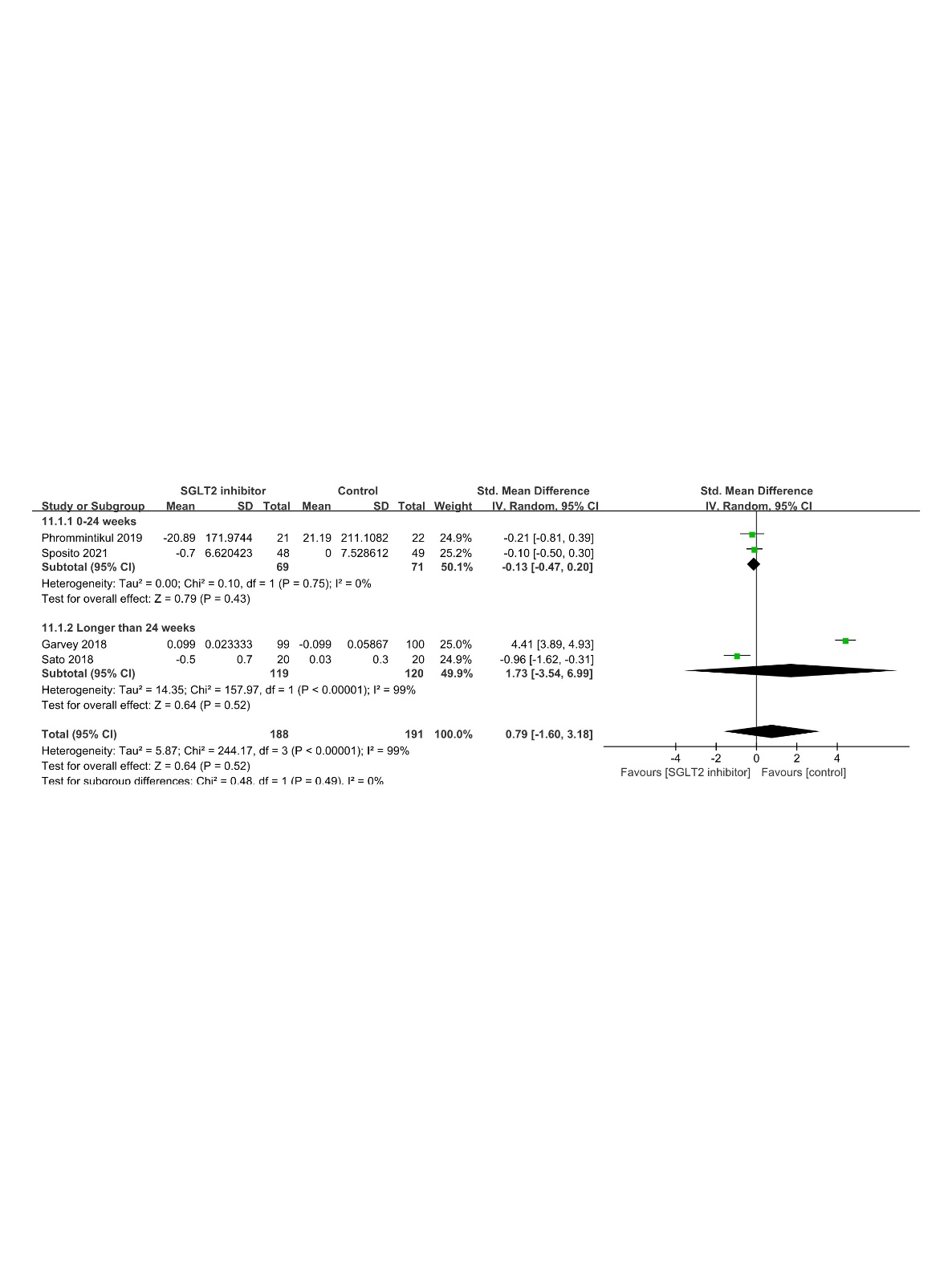


**(D) Adiponectin: SGLT2 inhibitors vs. placebo**


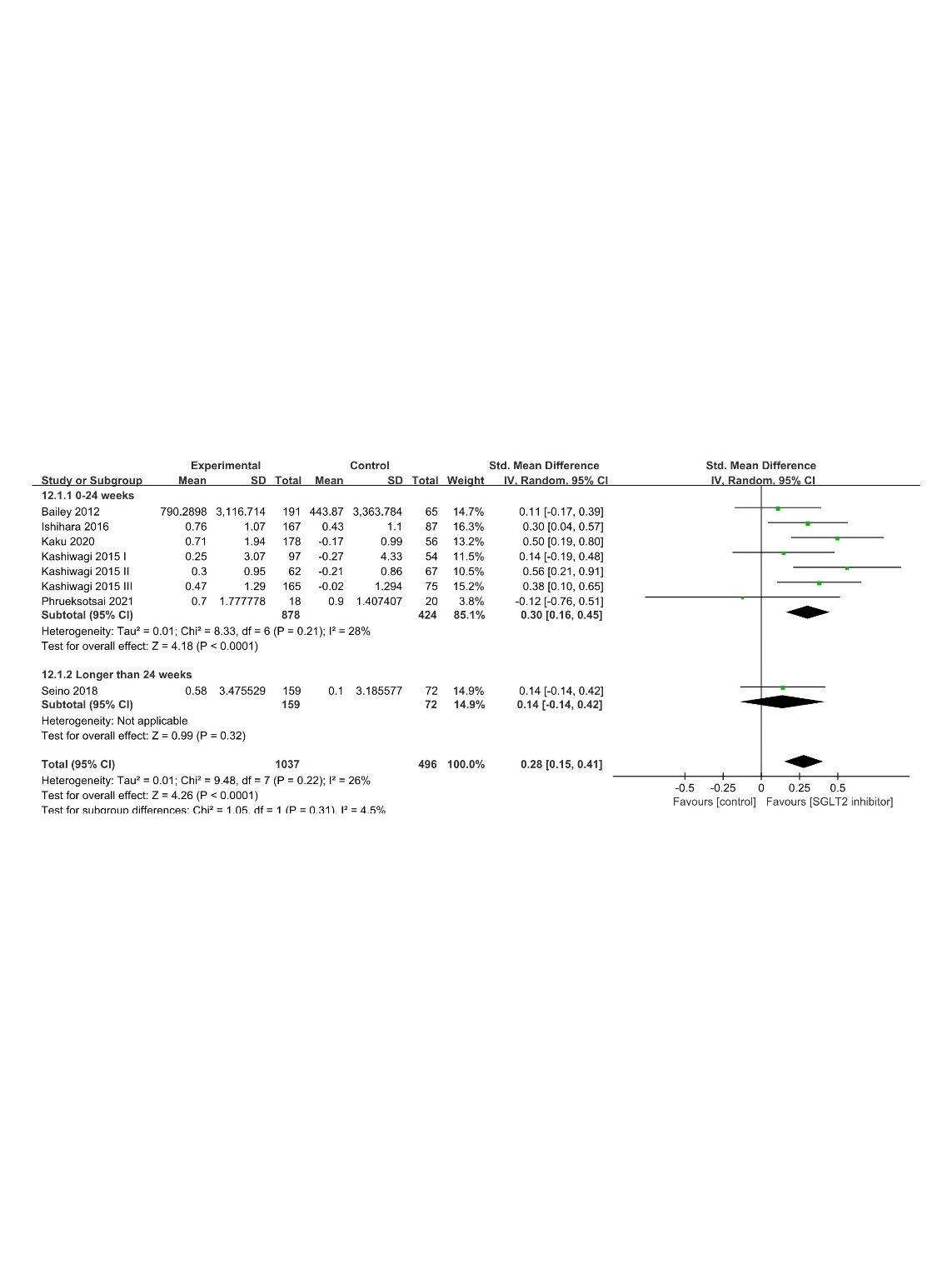


**(E) Adiponectin: SGLT2 inhibitors vs. diabetes medications**


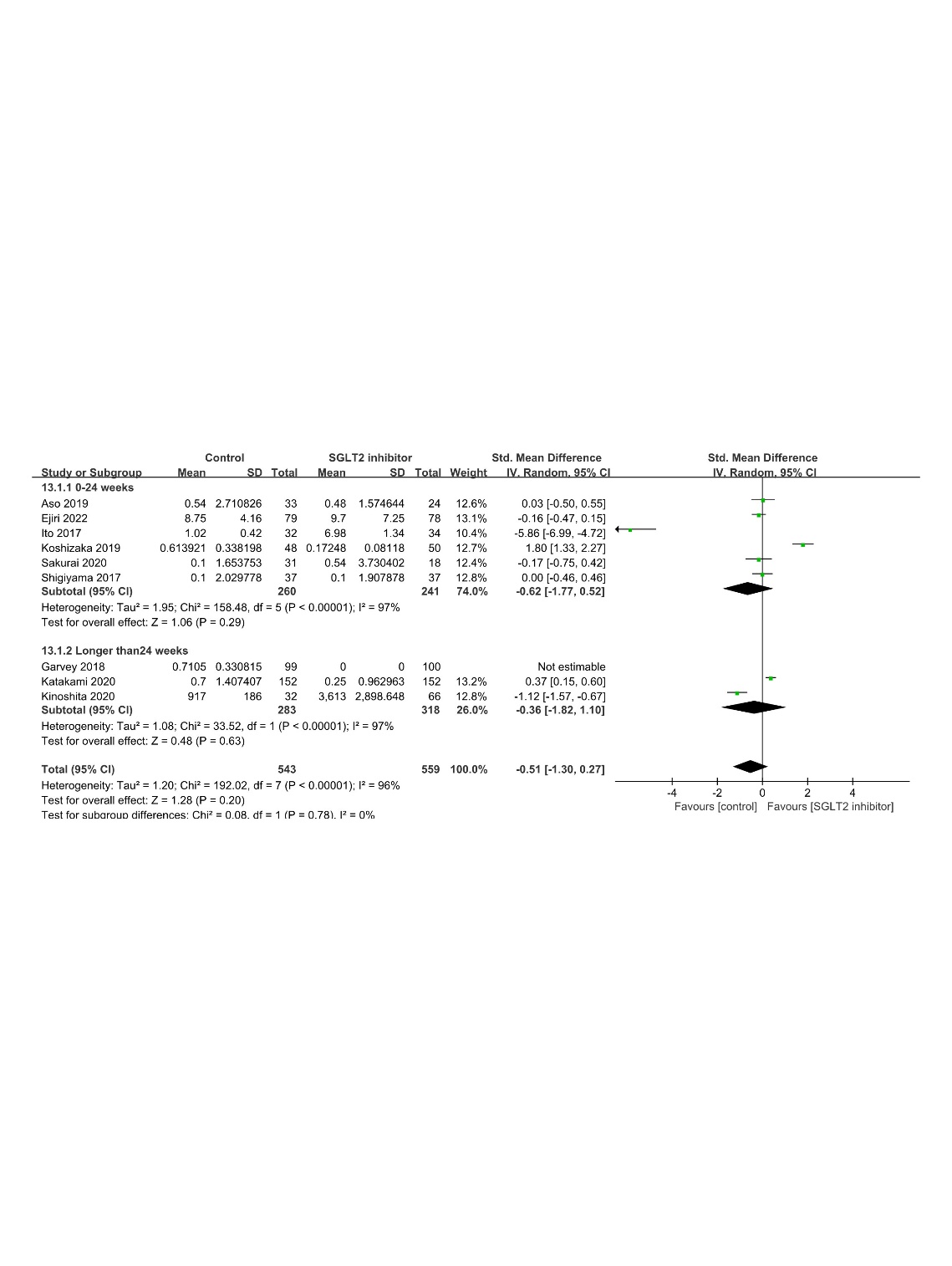


**(F) Leptin: SGLT2 inhibitors vs. placebo**


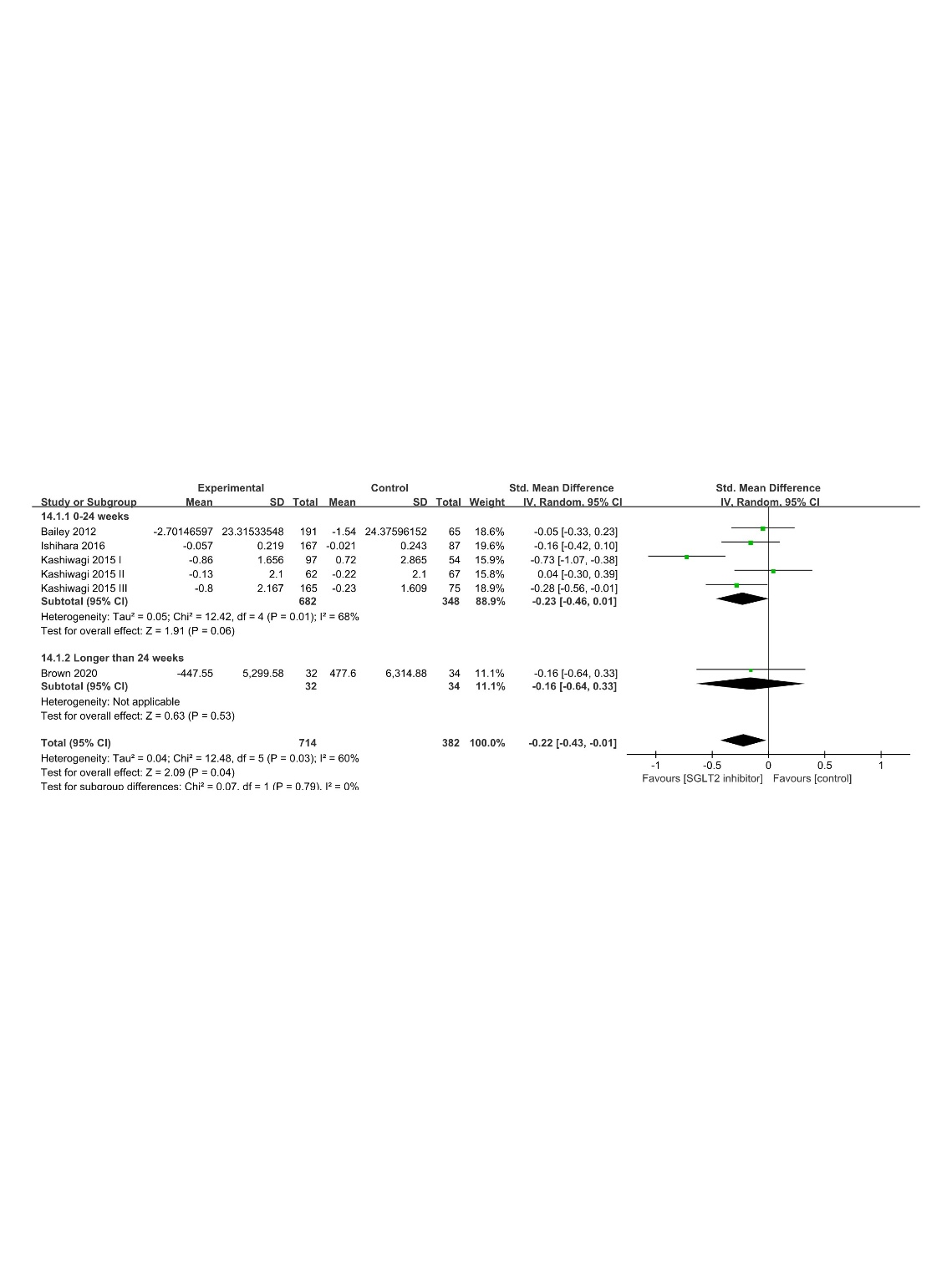


**(G) Leptin: SGLT2 inhibitors vs. diabetes medications**


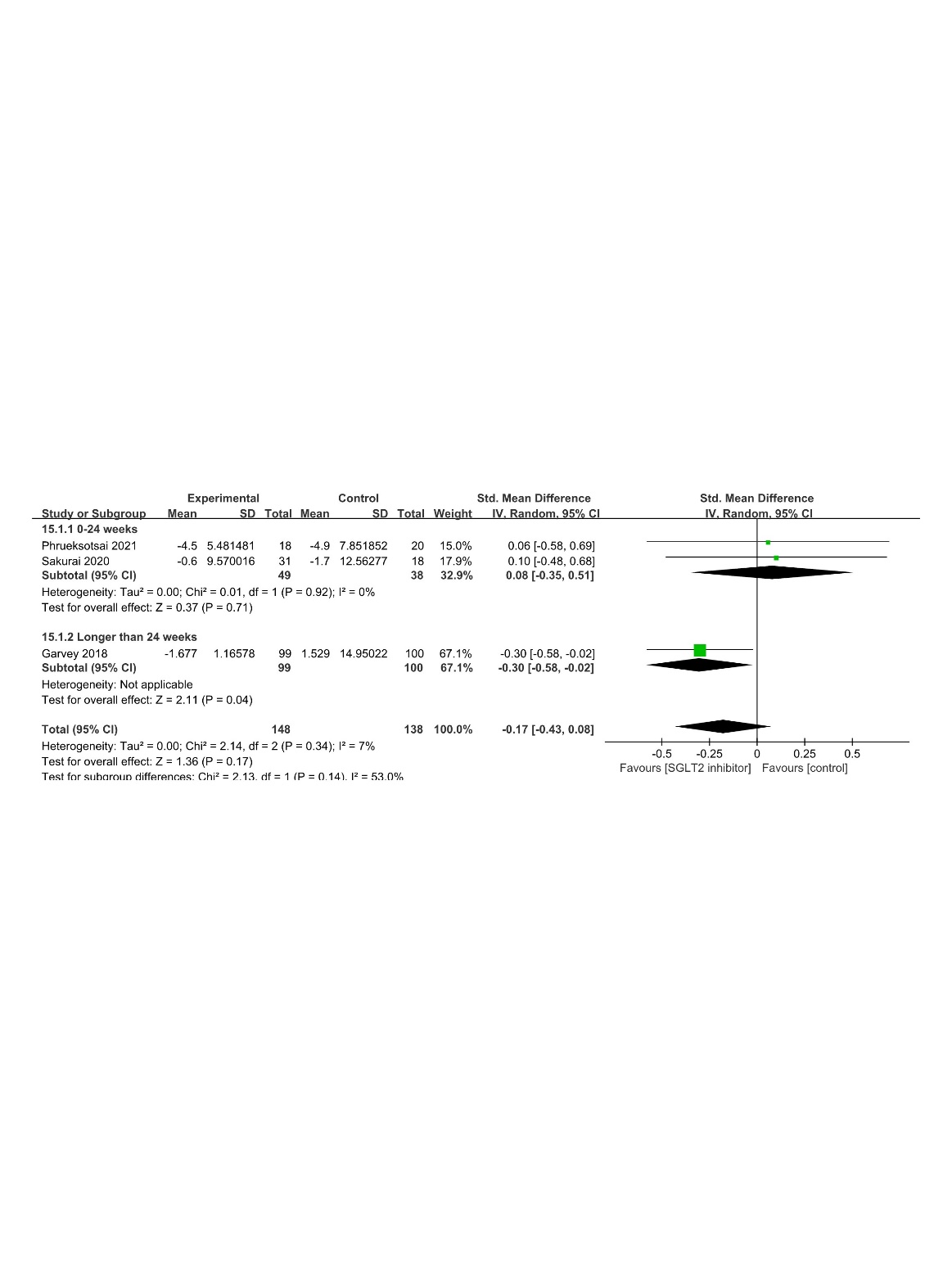


**Figure S3. Funnel plot of all eligible studies.**

1. **C-reactive protein**


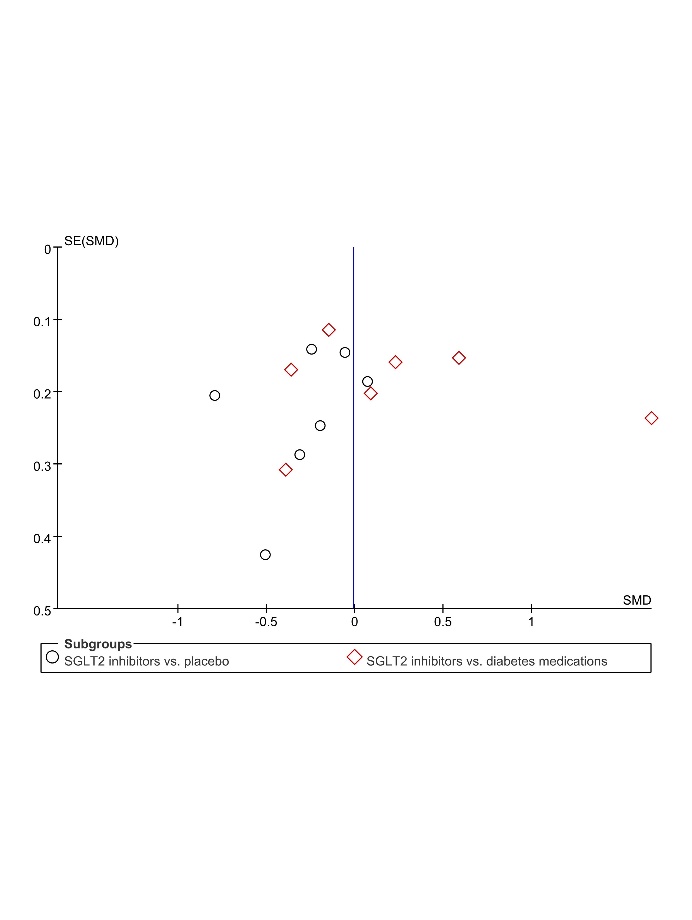


1. **Tumor necrosis factor-alpha**


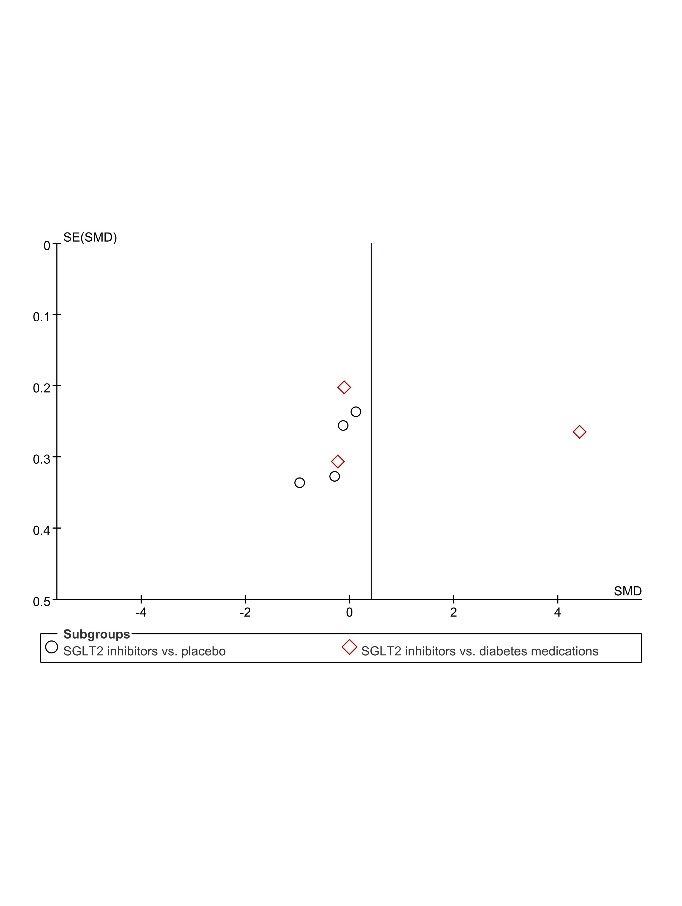


1. **Interleukin-6**


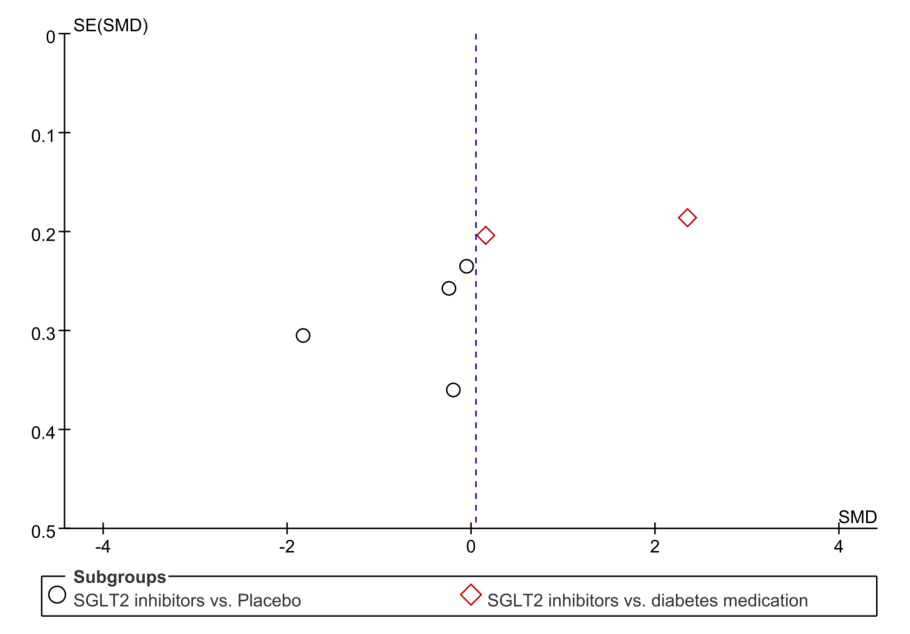


1. **Adiponectin**


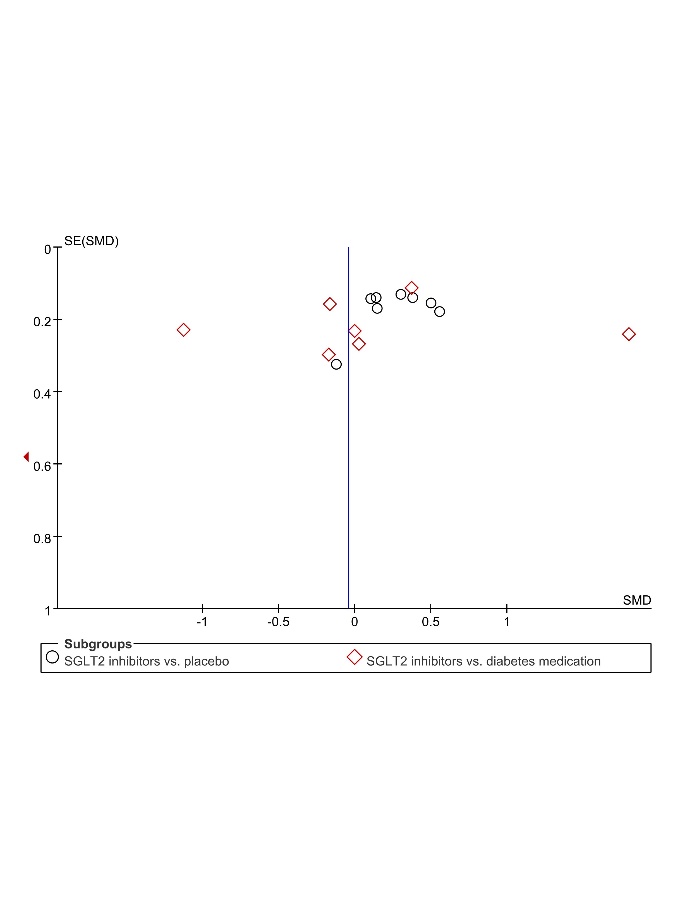


1. **Leptin**

**
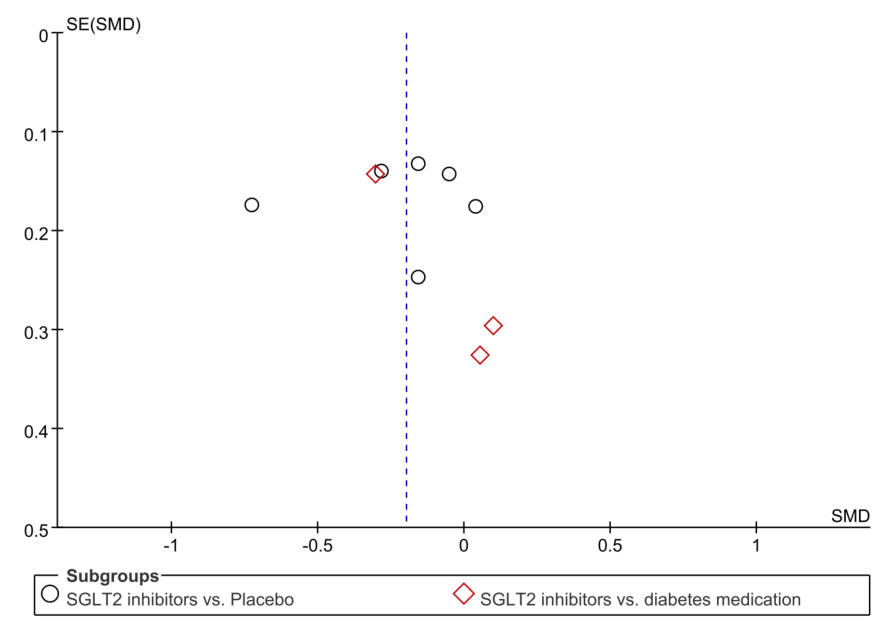
**

1. **Ferritin**

**
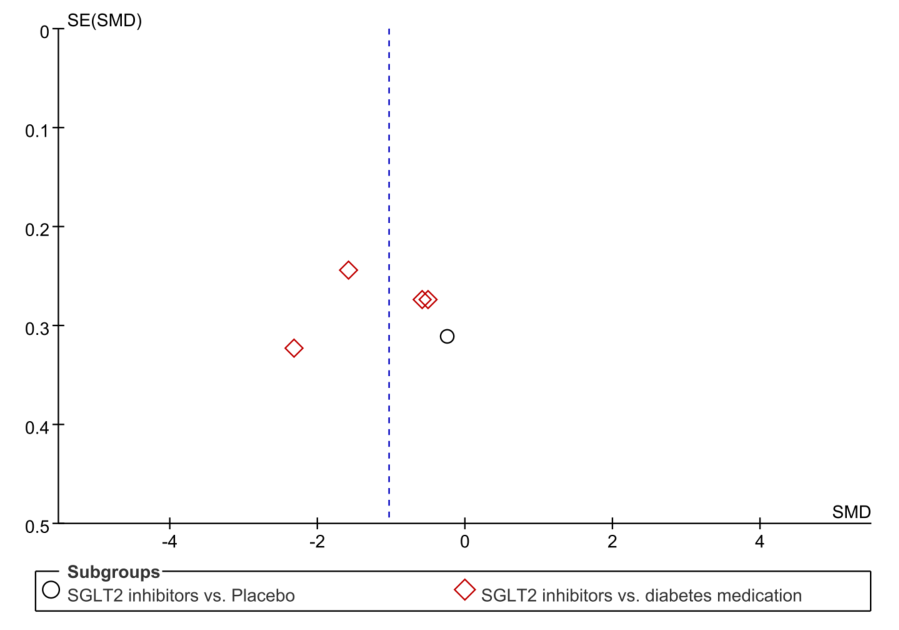
**

1. **Plasminogen activator inhibitor-1**

**
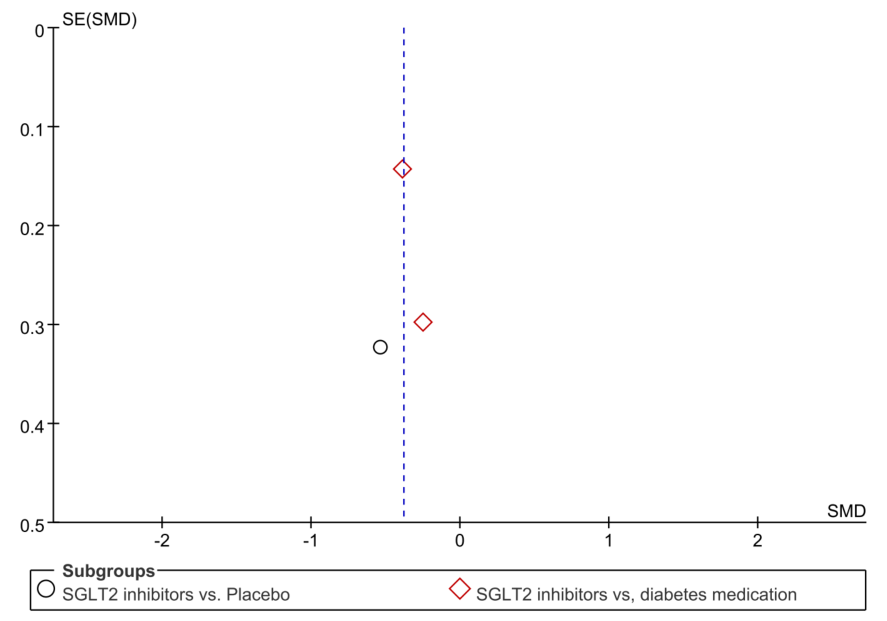
**

## Supplementary Tables

**Table S1. PRISMA 2020 checklist.**

| **Section and Topic** | **Item #** | **Checklist item** | **Location where item is reported** |
| --- | --- | --- | --- |
| **TITLE** | | |  |
| Title | 1 | Identify the report as a systematic review. | Page 1, line 1-2 |
| **ABSTRACT** | | |  |
| Abstract | 2 | See the PRISMA 2020 for Abstracts checklist. | Page 1-2, line 17-39 |
| **INTRODUCTION** | | |  |
| Rationale | 3 | Describe the rationale for the review in the context of existing knowledge. | Page 3, line 41-69 |
| Objectives | 4 | Provide an explicit statement of the objective(s) or question(s) the review addresses. | Page 3, line 69-70 |
| **METHODS** | | |  |
| Eligibility criteria | 5 | Specify the inclusion and exclusion criteria for the review and how studies were grouped for the syntheses. | Page 3, line 82-89 |
| Information sources | 6 | Specify all databases, registers, websites, organisations, reference lists and other sources searched or consulted to identify studies. Specify the date when each source was last searched or consulted. | Page 3, line 72-81 |
| Search strategy | 7 | Present the full search strategies for all databases, registers and websites, including any filters and limits used. | Page 3, line 72-81 |
| Selection process | 8 | Specify the methods used to decide whether a study met the inclusion criteria of the review, including how many reviewers screened each record and each report retrieved, whether they worked independently, and if applicable, details of automation tools used in the process. | Page 3, line 90-94 |
| Data collection process | 9 | Specify the methods used to collect data from reports, including how many reviewers collected data from each report, whether they worked independently, any processes for obtaining or confirming data from study investigators, and if applicable, details of automation tools used in the process. | Page 3, line 96-105 |
| Data items | 10a | List and define all outcomes for which data were sought. Specify whether all results that were compatible with each outcome domain in each study were sought (e.g. for all measures, time points, analyses), and if not, the methods used to decide which results to collect. | Page 3, line 90-105 |
|  | 10b | List and define all other variables for which data were sought (e.g. participant and intervention characteristics, funding sources). Describe any assumptions made about any missing or unclear information. | Page 3, line 97-99 |
| Study risk of bias assessment | 11 | Specify the methods used to assess risk of bias in the included studies, including details of the tool(s) used, how many reviewers assessed each study and whether they worked independently, and if applicable, details of automation tools used in the process. | Page 3, line 100-103 |
| Effect measures | 12 | Specify for each outcome the effect measure(s) (e.g. risk ratio, mean difference) used in the synthesis or presentation of results. | Page 3, line 107-115 |
| Synthesis methods | 13a | Describe the processes used to decide which studies were eligible for each synthesis (e.g. tabulating the study intervention characteristics and comparing against the planned groups for each synthesis (item #5)). | Page 3, line 107-115 |
|  | 13b | Describe any methods required to prepare the data for presentation or synthesis, such as handling of missing summary statistics, or data conversions. | Page 3, line 107-115 |
|  | 13c | Describe any methods used to tabulate or visually display results of individual studies and syntheses. | Page 3, line 107-115 |
|  | 13d | Describe any methods used to synthesize results and provide a rationale for the choice(s). If meta-analysis was performed, describe the model(s), method(s) to identify the presence and extent of statistical heterogeneity, and software package(s) used. | Page 3, line 107-115 |
|  | 13e | Describe any methods used to explore possible causes of heterogeneity among study results (e.g. subgroup analysis, meta-regression). | Page 3, line 107-115 |
|  | 13f | Describe any sensitivity analyses conducted to assess robustness of the synthesized results. | Page 3, line 107-115 |
| Reporting bias assessment | 14 | Describe any methods used to assess risk of bias due to missing results in a synthesis (arising from reporting biases). | Page 3, line 107-115 |
| Certainty assessment | 15 | Describe any methods used to assess certainty (or confidence) in the body of evidence for an outcome. | Page 3, line 107-115 |
| **RESULTS** | | |  |
| Study selection | 16a | Describe the results of the search and selection process, from the number of records identified in the search to the number of studies included in the review, ideally using a flow diagram. | Page 4, line 119-129 |
|  | 16b | Cite studies that might appear to meet the inclusion criteria, but which were excluded, and explain why they were excluded. | Page 5, line 184-185 |
| Study characteristics | 17 | Cite each included study and present its characteristics. | Page 4, line 119-129 |
| Risk of bias in studies | 18 | Present assessments of risk of bias for each included study. | Page 4, line 131-137 |
| Results of individual studies | 19 | For all outcomes, present, for each study: (a) summary statistics for each group (where appropriate) and (b) an effect estimate and its precision (e.g. confidence/credible interval), ideally using structured tables or plots. | Page 4-6, line 138-201 |
| Results of syntheses | 20a | For each synthesis, briefly summarise the characteristics and risk of bias among contributing studies. | Page 4-6, line 138-201 |
|  | 20b | Present results of all statistical syntheses conducted. If meta-analysis was done, present for each the summary estimate and its precision (e.g. confidence/credible interval) and measures of statistical heterogeneity. If comparing groups, describe the direction of the effect. | Page 4-6, line 138-201 |
|  | 20c | Present results of all investigations of possible causes of heterogeneity among study results. | Page 4-6, line 138-201 |
|  | 20d | Present results of all sensitivity analyses conducted to assess the robustness of the synthesized results. | Page 4-6, line 138-201 |
| Reporting biases | 21 | Present assessments of risk of bias due to missing results (arising from reporting biases) for each synthesis assessed. | Page 4, line 131-137 |
| Certainty of evidence | 22 | Present assessments of certainty (or confidence) in the body of evidence for each outcome assessed. | Page 4-6, line 138-201 |
| **DISCUSSION** | | |  |
| Discussion | 23a | Provide a general interpretation of the results in the context of other evidence. | Page 6, line 203-214 |
|  | 23b | Discuss any limitations of the evidence included in the review. | Page 7, line 257-275 |
|  | 23c | Discuss any limitations of the review processes used. | Page 7, line 257-275 |
|  | 23d | Discuss implications of the results for practice, policy, and future research. | Page 7, line 281-283 |
| **OTHER INFORMATION** | | |  |
| Registration and protocol | 24a | Provide registration information for the review, including register name and registration number, or state that the review was not registered. | Not registered |
|  | 24b | Indicate where the review protocol can be accessed, or state that a protocol was not prepared. | Supplementary materials |
|  | 24c | Describe and explain any amendments to information provided at registration or in the protocol. | Not registered |
| Support | 25 | Describe sources of financial or non-financial support for the review, and the role of the funders or sponsors in the review. | Page 8, line 293-295 |
| Competing interests | 26 | Declare any competing interests of review authors. | Page 7, line 285-286 |
| Availability of data, code and other materials | 27 | Report which of the following are publicly available and where they can be found: template data collection forms; data extracted from included studies; data used for all analyses; analytic code; any other materials used in the review. | Page 8, line 297 |

**Table S2. Search strategies.**

We have searched PubMed, Cochrane Library, EMBASE, and Web of Science using the terms below, Pubmed for example (updated to October 12th, 2022):

| Search number | Query |
| --- | --- |
| 1 | "Sodium-Glucose Transporter 2 Inhibitors"[Mesh] |
| 2 | "SGLT2*" OR "SGLT-2*" OR "SGLT 2*" OR "Sodium glucose cotransporter 2 inhibitor*" OR "Sodium-glucose cotransporter 2 inhibitor*" |
| 3 | "Sodium-Glucose Transporter 2"[Mesh] |
| 4 | "Sodium-Glucose Transport Proteins"[Mesh] OR "SGLT*" OR "Sodium-Glucose Transport Protein*" OR "Sodium Glucose Transporter*" |
| 5 | "canagliflozin"[Title/Abstract] OR "dapagliflozin"[Title/Abstract] OR "empagliflozin"[Title/Abstract] OR "ertugliflozin"[Title/Abstract] OR "ipragliflozin"[Title/Abstract] OR "licogliflozin"[Title/Abstract] OR "remogliflozin etabonate"[Title/Abstract] OR "sergliflozin etabonate"[Title/Abstract] |
| 6 | #1 OR #2 OR #3 OR #4 OR #5 |
| 7 | "Inflammation"[Mesh] OR "Inflammation*" |
| 8 | "C-Reactive Protein"[Mesh] OR "C?Reactive Protein*" OR "hs*CRP" |
| 9 | "Tumor Necrosis Factor-alpha"[Mesh] OR "Tumor Necrosis Factor?alpha" OR "Cachectin" OR "TNF*alpha" OR "TNF Superfamily, Member 2" |
| 10 | "Interleukin-6"[Mesh] OR "Interleukin-6" OR "Interleukin 6" OR "IL6" OR "IL-6" |
| 11 | "Leptin"[Mesh] OR "Leptin" OR "Obese Protein" OR "Ob Protein" |
| 12 | "Adiponectin"[Mesh] OR "Adiponectin" OR "apM 1 Protein" OR "Adipocyte Complement Related Protein 30 kDa" |
| 13 | "Plasminogen Activator Inhibitor 1"[Mesh] OR "Plasminogen Activator Inhibitor 1" OR "PAI-1" OR "SERPINE1 Protein" OR "Serpin E1" |
| 14 | "Vascular Cell Adhesion Molecule-1"[Mesh] OR "Vascular Cell Adhesion Molecule-1" OR "Vascular Cell Adhesion Molecule 1" OR "VCAM-1" |
| 15 | "Ferritins"[Mesh] OR "Ferritins" OR "Isoferritin" |
| 16 | #7 OR #8 OR #9 OR #10 OR #11 OR #12 OR #13 OR #14 OR #15 |
| 17 | #6 AND #16 |
| 18 | (#17) NOT (review [Publication Type]) |
| 18 | #18 AND (Randomized Controlled Trial[Publication Type] OR Randomized Controlled Trial*[Title/Abstract] OR Clinical Trials[Title/Abstract] OR Randomized[Title/Abstract] OR Randomized Clinical[Title/Abstract] OR Trials[Title/Abstract]) |
